# Guidelines for whole genome bisulphite sequencing of intact and FFPET DNA on the Illumina HiSeq X Ten

**DOI:** 10.1186/s13072-018-0194-0

**Published:** 2018-05-28

**Authors:** Shalima S. Nair, Phuc-Loi Luu, Wenjia Qu, Madhavi Maddugoda, Lily Huschtscha, Roger Reddel, Georgia Chenevix-Trench, Martina Toso, James G. Kench, Lisa G. Horvath, Vanessa M. Hayes, Phillip D. Stricker, Timothy P. Hughes, Deborah L. White, John E. J. Rasko, Justin J.-L. Wong, Susan J. Clark

**Affiliations:** 1 Genomics and Epigenetics Division, Garvan Institute of Medical Research, Darlinghurst, NSW 2010 Australia; 2 St Vincent’s Clinical School, UNSW, Sydney, NSW 2010 Australia; 3 Cancer Research Unit, Children’s Medical Research Institute, University of Sydney, Westmead, NSW 2145 Australia; 4 QIMR Berghofer, Brisbane, QLD 4006 Australia; 5 Department of Tissue Pathology and Diagnostic Oncology, Royal Prince Alfred Hospital, Camperdown, NSW Australia; 6 Central Clinical School, Sydney Medical School, University of Sydney, Camperdown, NSW Australia; 7 Clinical Prostate Cancer Research, The Kinghorn Cancer Centre, Garvan Institute of Medical Research, Darlinghurst, NSW Australia; 8 Chris O’Brien Lifehouse, Camperdown, NSW Australia; 9 Department of Urology, St. Vincent’s Hospital, Darlinghurst, NSW Australia; 10 Cancer Theme, South Australian Health and Medical Research Institute, Adelaide, SA Australia; 11 Australian Leukaemia and Lymphoma Group, Melbourne, Australia; 12 Discipline of Medicine, University of Adelaide, Adelaide, SA Australia; 13 Department of Haematology, SA Pathology, Adelaide, SA Australia; 14 Faculty of Health Science and Faculty of Science, University of Adelaide, Adelaide, SA Australia; 15 Australian Genomic Health Alliance, Melbourne, Australia; 16 Gene and Stem Cell Therapy Program, Centenary Institute, University of Sydney, Camperdown, NSW 2050 Australia; 17 Sydney Medical School, University of Sydney, Sydney, NSW 2006 Australia; 18 Cell and Molecular Therapies, Royal Prince Alfred Hospital, Camperdown, 2050 Australia; 19 Gene Regulation in Cancer Laboratory, Centenary Institute, University of Sydney, Camperdown, NSW 2050 Australia; 20 Epigenetics Research Program, The Garvan Institute of Medical Research, 384 Victoria St, Darlinghurst, Sydney, NSW 2010 Australia

**Keywords:** DNA methylation, Whole genome bisulphite sequencing, HiSeq X Ten, HiSeq 2500, Epigenetics, SNP

## Abstract

**Background:**

Comprehensive genome-wide DNA methylation profiling is critical to gain insights into epigenetic reprogramming during development and disease processes. Among the different genome-wide DNA methylation technologies, whole genome bisulphite sequencing (WGBS) is considered the gold standard for assaying genome-wide DNA methylation at single base resolution. However, the high sequencing cost to achieve the optimal depth of coverage limits its application in both basic and clinical research. To achieve 15× coverage of the human methylome, using WGBS, requires approximately three lanes of 100-bp-paired-end Illumina HiSeq 2500 sequencing. It is important, therefore, for advances in sequencing technologies to be developed to enable cost-effective high-coverage sequencing.

**Results:**

In this study, we provide an optimised WGBS methodology, from library preparation to sequencing and data processing, to enable 16–20× genome-wide coverage per single lane of HiSeq X Ten, HCS 3.3.76. To process and analyse the data, we developed a WGBS pipeline (METH10X) that is fast and can call SNPs. We performed WGBS on both high-quality intact DNA and degraded DNA from formalin-fixed paraffin-embedded tissue. First, we compared different library preparation methods on the HiSeq 2500 platform to identify the best method for sequencing on the HiSeq X Ten. Second, we optimised the PhiX and genome spike-ins to achieve higher quality and coverage of WGBS data on the HiSeq X Ten. Third, we performed integrated whole genome sequencing (WGS) and WGBS of the same DNA sample in a single lane of HiSeq X Ten to improve data output. Finally, we compared methylation data from the HiSeq 2500 and HiSeq X Ten and found high concordance (Pearson *r* > 0.9×).

**Conclusions:**

Together we provide a systematic, efficient and complete approach to perform and analyse WGBS on the HiSeq X Ten. Our protocol allows for large-scale WGBS studies at reasonable processing time and cost on the HiSeq X Ten platform.

**Electronic supplementary material:**

The online version of this article (10.1186/s13072-018-0194-0) contains supplementary material, which is available to authorized users.

## Background

DNA methylation plays an important role in differentiation and development [1, 2]. Alterations in DNA methylation patterns are associated with various human diseases, including cancer and diabetes [3–5]. DNA methylation is the covalent addition of methyl groups to DNA. In mammals, methyl groups are most commonly added to a ‘CpG site’, which is a cytosine (C) base that is immediately adjacent to guanine (G) base. There are ~ 28 million CpG sites in the human genome, and 70–80% are methylated in normal, healthy cells. The remaining unmethylated sites are found in clusters that are often 1000 bp long, termed CpG islands [6]. CpG islands are predominately located at gene promoters, and these genes are typically expressed and include almost all the housekeeping genes present in the human genome [7, 8]. In cancer, CpG island promoters are prone to hypermethylation and associated genes silencing. In contrast the bulk of the genome in cancer is subject to hypomethylation and gene activation of cancer-associated oncogenes [9, 10].

To enable comprehensive methylation studies to be performed across large-scale clinical cohorts in order to profile methylation changes as we age or to identify changes that occur with disease progression, we need cost-saving advances in methylation sequencing technologies. To date, several methods have been developed to perform DNA methylation analysis on a genome-wide scale. These can be divided into three broad categories: (1) enrichment based, either using antibodies, restriction enzymes or by immuno-precipitation followed by sequencing [11–14], (2) bisulphite conversion followed by sequencing [1, 15] and (3) array-based methods, such as Illumina arrays [16–19]. Among these, bisulphite conversion followed by next-generation sequencing is known as the best approach to provide the complete human methylome [20–22]. Whole genome bisulphite sequencing (WGBS) maps cytosine methylation across the entire genome at single base resolution. WGBS is currently the standard method of choice for studies that generate reference methylomes [23–25]. In addition, WGBS is also increasingly used in basic and clinical research [26]. Although WGBS has been widely accepted as the gold standard method to assay genome-wide DNA methylation, the high cost [22] and depth of sequencing required [27] make it a challenge for large-scale DNA methylation studies. Ever since the time WGBS was developed in 2009 the number of large-scale WGBS studies is limited [28, 29], either small numbers of samples are studied at a high coverage [15, 30] or larger numbers of samples are studied at low coverage [31]. One of the earliest publications on WGBS was performed at 30× coverage in order to compare the DNA methylation profiles of an embryonic stem cell and a fibroblast cell line [15]. Here, 376 lanes of bisulphite sequencing were performed on the Illumina GAII instrument to achieve this coverage. Similarly, Kulis et al. [30] performed WGBS on sorted B cells at different stages of differentiation at a depth of 54× for 12 biological samples on at least 85 lanes of the HiSeq 2000. Large-scale methylome studies may not therefore be affordable to many researchers due to the high cost required to run multiple lanes. The advent of the HiSeq X Ten has opened up possibilities of generating WGBS data with better coverage per lane of sequencing making it potentially more cost-effective.

Despite the promising potential of performing methylome studies on the HiSeq X Ten, achieving optimum coverage of WGBS is challenging. HiSeq X Ten has a fixed sequencing length of 300 cycles (150 bp paired end). Therefore, to achieve maximum output with higher diversity from this platform, the library needs to be at least 300 bp long—excluding the length of the adaptors on either side. Achieving this large library size for a WGBS library is difficult due to the fragmentation process during bisulphite conversion [32]. In addition, since bisulphite-converted libraries comprise an unbalanced base composition, it is a challenge to achieve optimal cluster passing filter of the library without balancing the library with another library of uniform base composition. However, the fast EXamp amplification chemistry of the HiSeq X Ten can result in preferential amplification and cluster formation of smaller insert size libraries. Therefore, even a 5% contamination of adaptor dimers in the library can result in up to 60% of the sequencing output being adaptor sequence. In addition, a higher loading concentration of the bisulphite library can lead to polyclonal clusters and a lower loading concentration can lead to higher duplicate reads.

In this study, we optimised and developed a working protocol for the preparation and processing of WGBS data prepared from good quality DNA and FFPET material to maximise data output from the HiSeq X Ten (Additional file 2: Fig. 5a, b). We provide guidelines on the best method to achieve larger bisulphite library size and optimum loading concentration for the bisulphite library and the spike-in library, for the HiSeq X Ten, HCS 3.3.76. We consistently achieve ~ 16–20× coverage per lane of WGBS data for the good quality DNA and ~ 10–14× per lane for FFPET. Finally, we explored the possibility of performing integrated WGS and WGBS from the same DNA samples in the same lane of the HiSeq X Ten platform and show that this results in minimal read wastage. Higher methylome coverage using the HiSeq X Ten platform now enables larger-scale population-based WGBS studies to be potentially more cost-effective.

## Results

### Comparison of library sequencing preparation methods using intact genomic DNA with the Illumina HiSeq 2500 platform

To optimise the coverage output per lane from WGBS, we first compared different library preparation methods on the HiSeq 2500 on a rapid run (two lanes) to determine differences in library fragment size distribution. We compared two pre-bisulphite (pre-BS) library preparation methods, where adaptor tagging and ligation are performed *before* bisulphite conversion, and three post-bisulphite (post-BS) methods, where adaptor tagging and ligation are performed *after* bisulphite conversion [33] (‘Methods’ section). The pre-BS library preparation methods used were KAPA LTP and KAPA Hyperprep, from KAPA Biosystems, with input amounts of, 1000 and 100 ngs, respectively. The post-BS methods we used were TruSeq DNA methylation kit from Illumina, TruMethyl WG from Cambridge Epigenetix and Accel-NGS Methyl-Seq from Swift Biosciences, with input amounts of, 50, 200 and 100 ngs DNA, respectively. In addition to the original methods from the manufacturer, we also modified the AMpure XP bead ratios during the size selection step of the library preparation to determine if a change in bead ratios had an effect on the library fragment size and subsequent overall sequencing coverage across the genome. Details of the modifications are provided (‘Methods’ section). For consistency, the libraries were prepared from the same cell line (LNCaP) DNA sample.

**Fig. 1:**
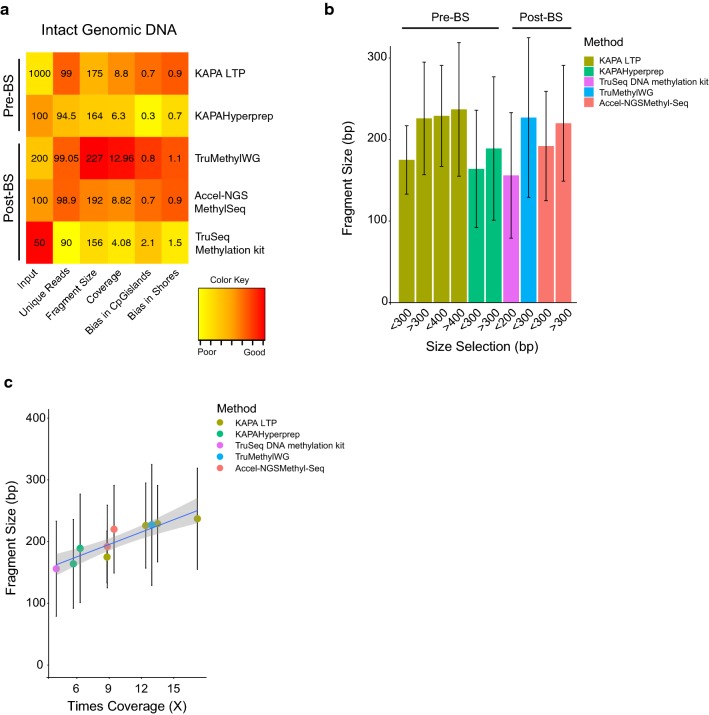
Comparing different library preparation methods using genomic DNA on the HiSeq 2500. **a** Cluster plot of sequencing output metrics obtained from different library preparation methods from intact genomic DNA. **b** Bar graph showing fragment size distribution for different bead size selection for each library preparation method. **c** Plot with *x*-axis indicating coverage and *y*-axis indicating fragment size illustrating how increase in fragment size leads to improved coverage

Table 1 summarises the differences observed in percentage of duplicate reads, library fragment size distribution, genome-wide coverage and the ratio of bias in coverage across CpG islands and CpG shores (illustrated in Fig. 1a). Details of how the bias ratio was estimated are provided in the methods section. First we found that the library preparation methods with altered size selections (< 300 and > 400 bp) gave rise to different library fragment size distribution (Table 1 and Fig. 1b) and associated differences in genomic coverage. For example, the KAPA LTP kit < 300 bp size selection resulted in average library fragment size 175 bp and 8.8× per lane of sequencing whereas > 400 bp size selection resulted in average library fragment size 237 bp and 17.2× per lane of sequencing (Table 1). The trend for larger library fragment size to improve genomic coverage was observed across all the different library preparation methods (Fig. 1c). Second, we observed that, among the manufacturer’s recommended methods, both the KAPA LTP and KAPA Hyperprep result in an under-representation of reads across CpG islands (Additional file 2: Fig. 1a, b), whereas the TruSeq DNA methylation method shows a bias towards CpG islands (Additional file 2: Fig. 1c). The TruMethyl WG showed the least bias (0.8 CpG islands/1.1 CpG shores), with almost equal coverage across CpG islands, CpG shores and other regions of the genome (7.68×, 10.80×, 10.08×) closely followed by the Accel-NGS-Methyl-Seq method (Additional file 2: Fig. 1d, e). Based on these combined results for coverage and bias, we decided to further optimise the TruMethyl WG library method for WGBS of intact DNA on the HiSeq X Ten platform.

**Table 1:** Comparison of different library preparation kits on intact genomic DNA using HiSeq 2500 Platform

Library preparation method	Library preparation kit	Input amount (ng)	Size selection (bp)	Duplicate read (%)	Fragment size (stdev)	Coverage^b^	Ratio of bias in CpG island/shores
Pre-BS	KAPA LTP (Kapa Biosystems)	1000	< 300^a^	1.00	175 bp (42 bp)	8.8×	0.7:0.9
			> 300	3.50	226 bp (69 bp)	12.4×	0.3:0.7
			< 400	0.72	229 bp (62 bp)	13.5×	0.3:0.6
			> 400	1.90	237 bp (82 bp)	17.2×	0.8:0.9
	KAPA Hyperprep (Kapa Biosystems)	100	< 300^a^	5.50	164 bp (72 bp)	6.3×	0.3:0.7
			> 300	5.50	189 bp (88 bp)	7.0×	0.3:0.7
Post-BS	TruSeq DNA methylation (Illumina)	50	< 200^a^	10.00	156 bp (77 bp)	4.0×	2.1:1.5
	TruMethyl WGv 1.9 (Cambridge Epigenetix)	200	> 300^a^	0.95	227 bp (98 bp)	12.9×	0.8:1.1
	Accel-NGS Methyl-Seq (Swift Biosciences)	100	< 300^a^	1.40	179 bp (61 bp)	10.7×	0.7:0.9
			> 300	1.10	202 bp (70 bp)	12.6×	0.7:0.9

^a^Manufacturer’s recommended method

^b^Two lanes( HiSeq 2500 rapid run)

### Optimisation of library loading concentration and spike-in balanced libraries on the HiSeq X Ten

**Fig. 2:**
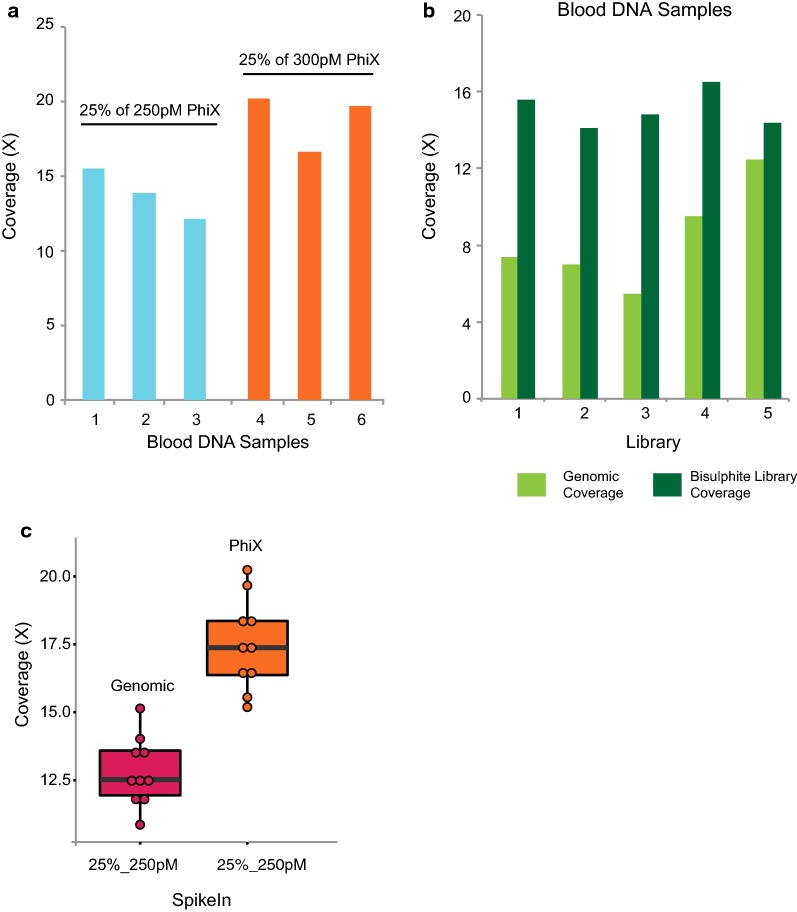
Optimisation of spike-in library and its loading concentration. **a** Bar plot showing the difference in coverage obtained from sequencing two different loading concentrations of the PhiX spike-in library for six WGBS libraries obtained from blood DNA samples. **b** Bar plot showing the coverage obtained from both the bisulphite library and genomic library when 25% of genomic library is spiked in instead of PhiX, for five different bisulphite libraries prepared by the TruMethyl WG method. **c** Box plot showing the coverage distribution of the same set of bisulphite libraries when spiked with either 25% of 250 pM genomic library or 25% of 300 pM of PhiX library

A challenge in performing WGBS on the HiSeq X Ten is the unbalanced base composition of the bisulphite library, and therefore, the bisulphite-treated DNA requires spiking with DNA of a normal base composition in order to maximise the cluster passing filter during sequencing. For whole genome sequencing (WGS), the optimal loading concentration of library recommended by Illumina is 300 pM. Therefore, we used 300 pM as the loading concentration for the WGBS library (Table 2), cell line (LNCaP DNA). To balance the nucleotide composition of the bisulphite library, we spiked in PhiX (from Illumina) at 25% of 250 pM and obtained a coverage of 8.7× from one lane of sequencing on the HiSeq X Ten (Table 2). To further improve the coverage, we lowered the loading concentration of the bisulphite library to 250 pM and performed sequencing with two different loading concentration of PhiX spike-in, namely 250 and 300 pM. This improved coverage from 8.70× to 15.19× using the same cell line input DNA (Table 2). To further test which of the PhiX loading concentration gave a higher coverage, we compared the sequencing coverage output from six different DNA samples isolated from blood, three of which were spiked with 25% of 250 pM PhiX and the other three were spiked with 25% of 300 pM PhiX spike (Table 2, blood DNA samples). We observed that the loading concentration of 250 pM for the bisulphite library with a combination of 300 pM loading concentration for PhiX spiked at a percentage of 25% gave a marked and consistent increase in overall coverage of the bisulphite genome (16.59–20.24×) (Fig. 2a).

**Table 2:** Comparison of coverage obtained with different bisulphite library and PhiX loading concentrations

Library preparation method	Library preparation kit name	Input amount (ng)	DNA samples	Sequencing platform	Bisulphite library loading concentration	PhiX loading concentration	% of spike-in	Coverage/lane
Post-BS	TruMethyl WG v1.9	200	Cell line (LNCaP)	HiSeq X Ten	300 pM	250 pM	25%	8.70×
			Cell line (LNCaP)		250 pM	250 pM		14.50×
			Cell line (LNCaP)			300 pM		15.19×
			1. Blood sample (2)			250 pM		15.46×
			2. Blood sample (3)			250 pM		13.87×
			3. Blood sample (4)			250 pM		12.15×
			4. Blood sample (6)			300 pM		20.24×
			5. Blood sample (23)			300 pM		16.59×
			6. Blood sample (24)			300 pM		19.67×

However, the problem with using PhiX DNA to spike is that at least 25% of the sequencing reads are ‘wasted’. Therefore, to maximise the sequencing information we tested the effect of using genomic DNA (cell line DNA) as the balanced DNA control spike. First we spiked in 25% of 250 pM of unconverted genomic library DNA (similar size to the matching bisulphite libraries) to 5 independent libraries prepared from cell line DNA and DNA from blood (Table 3). We obtained bisulphite genome mean coverage of ~ 15× per lane of the HiSeq X Ten and the whole genome mean coverage of ~ 8.4× per lane (Table 3; Fig. 2b). We next compared the coverage output between 25% of 300 pM PhiX and 25% of 250 pM genomic spike-in using the same set of 10 independent bisulphite libraries (blood DNA, cell line DNA) (Fig. 2c). Interestingly, we found that the best spike-in DNA to give maximum bisulphite coverage output is 25% of 300 pM PhiX, which resulted in 15.5×–20.2× WGBS coverage (Table 4). Therefore, if only methylome data are required, our results suggest that PhiX spike-in is preferable.

**Table 3:** Comparison of coverage from bisulphite and genomic library when 25% of genomic library is spiked

Library preparation method	Library preparation kit name	Input amount (ng)	WGBS library samples	Sequencing platform	WGBS library loading concentration	WGS library sample^a^	% of spike-in library	WGBS coverage/lane	WGS coverage/lane
Post-BS	TruMethyl WG v1.9	200	1. Cell line (LNCaP)	HiSeq X Ten	250 pM	Cell line (LNCaP)	25%	15.57×	7.40×
			2. Cell line (LNCaP)					14.10×	7.02×
			3. Cell line (B80-T17-P12)					16.50×	9.50×
			4. Blood sample 3					14.80×	5.47×
			5. Blood sample 4					14.40×	12.47×

^a^Genomic library used as spike-in

**Table 4:** Comparison of coverage and duplicate reads for bisulphite libraries when spiked with genomic library or PhiX

Library preparation method	Library preparation kit	Input amount (ng)	Sequencing platform	DNA samples	Coverage with genomic spike-in^a^	Coverage with PhiX spike-in^b^	Duplicate read % with genomic spike-in	Duplicate read % with PhiX spike-in
Post-BS	TruMethyl WG v1.9	200	HiSeq X Ten	1. Blood sample (23)	14.02×	16.6×	32	39
				2. Blood sample (24)	13.66×	19.67×	21	30
				3. Blood sample (25)	12.43×	18.37×	22	33
				4. Blood sample (26)	12.6×	17.34×	28	36
				5. Cell line (27)	12.36×	16.29×	33	44
				6. Cell line (28)	13.37×	18.33×	25	36
				7. Blood sample (5)	11.8×	15.19×	36	48
				8. Blood sample (6)	15.14×	20.24×	21	28
				9. Blood sample (13)	10.86×	17.41×	18	30
				10. Blood sample (14)	11.79×	15.55×	31	43

^a^25% of 250 pM genomic library spiked in

^b^25% of 300 pM PhiX library spiked in

### The potential for HiSeq X Ten to provide integrated WGS and WGBS on intact genomic DNA

**Fig. 3:**
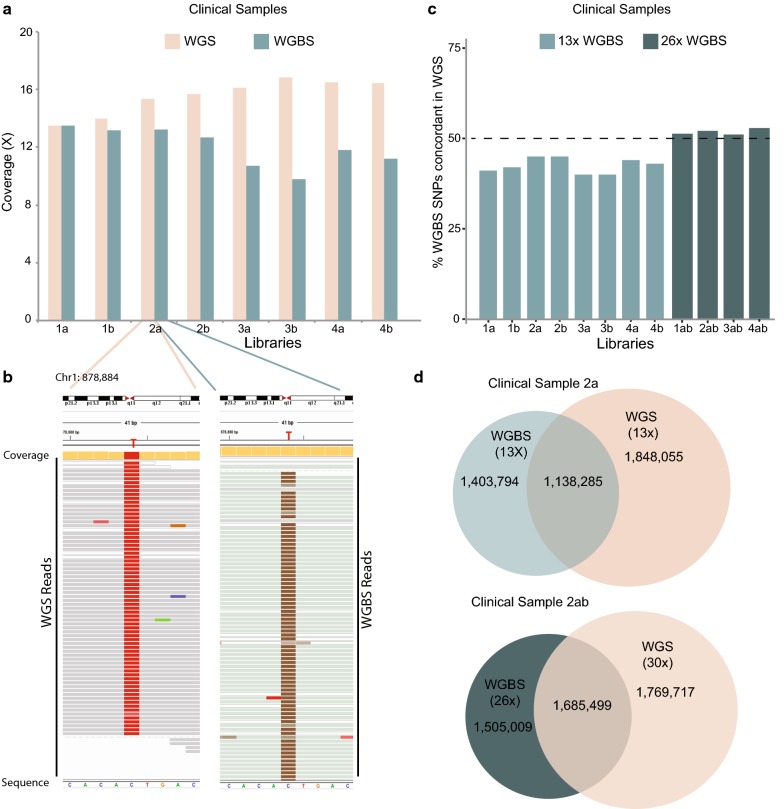
Integrating whole genome and whole genome bisulphite sequencing. **a** Bar plot depicting the coverage obtained when both genomic and its corresponding bisulphite library is sequenced on the same lane of the HiSeq X Ten, for four prostate cancer samples sequenced in duplicate (**a**, **b**). **b** A representative IGV plot showing a C to T SNP identified in both the WGS and WGBS data at approximately 13× coverage. **c** Bar plot indicating the percentage of SNPs from WGBS concordant in spike-in WGS at 13× and 26× coverage. **d** A representative Venn diagram for one prostate cancer sample, 2a showing the number of SNPs concordant and discordant at 13× and 26× coverage for both WGBS and spike-in WGS

To further capitalise on informative sequencing data per lane of sequencing, we tested the output reads from simultaneously sequencing the methylome and genome from the same DNA samples. We first tested spiking four independent bisulphite-converted libraries from clinical prostate cancer DNA samples (intact genomic DNA) with 250 pM of their matching genomic library in a 50:50 ratio. We performed the library prep and spike-in and sequencing on the HiSeq X Ten in technical duplicates (Table 5a, b) and observed that overall coverage per lane (for WGBS and WGS library together) is ~ 26× the library prep and spike, consisting of bisulphite library (~ 10–13× the library prep and spike) average coverage and matching genomic sequence coverage (~ 13–16× the library prep and spike) (Table 5; Fig. 3a). Using the Meth10X pipeline (see methods), we could detect exemplary SNPs from the WGBS data at coverage of 13× (Fig. 3b; Additional file 2: Fig. 2), which were confirmed in the WGS spike-in data of the same sample.

**Table 5:** Comparison of coverage from bisulphite and genomic library when sequenced in a 50:50 ratio on a single lane of HiSeq X Ten

Library preparation method	Library preparation kit name	Input amount (ng)	WGBS library sample	WGBS library loading concentration	WGS library sample^a^	% of spike-in library	WGBS coverage/lane	WGS coverage/lane
Post-BS	TruMethyl WG v1.9	200	1a. Prostate cancer DNA (5287)	250 pM	1a. Prostate cancer DNA (5287)	50%	13.48×	13.48×
			1b. Prostate cancer DNA (5287)		1b. Prostate cancer DNA (5287)		13.16×	13.97×
			2a. Prostate cancer DNA (5060)		2a. Prostate cancer DNA (5060)		13.20×	15.38×
			2b. Prostate cancer DNA (5060)		2b. Prostate cancer DNA (5060)		12.70×	15.70×
			3a. Prostate cancer DNA (13179)		3 a. Prostate cancer DNA (13179)		10.70×	16.12×
			3b. Prostate cancer DNA (13179)		3b. Prostate cancer DNA (13179)		9.80×	16.83×
			4a. Prostate cancer DNA (10738)		4a. Prostate cancer DNA (10738)		11.80×	16.50×
			4b. Prostate cancer DNA (10738)		4b. Prostate cancer DNA (10738)		11.20×	16.45×

^a^50% of corresponding genomic library used as spike-in

Approximately 40–45% of SNPs called from WGBS (~ 10–13×) coverage data were found to be concordant with the SNPs identified from spike-in WGS (~ 13–16×) coverage data for each sample tested, (Fig. 3c, d; Additional file 1: Table 1). At higher coverage (~ 20–26×), the number of SNPs commonly called between WGBS and spike-in WGS data increased to 51–53% (Fig. 3c, d; Additional file 1: Table 1). To compare the fidelity of SNPs called from WGBS and WGS spike-in data, we first identified the overlap of variants called in two single lanes of HiSeq X Ten sequencing (each lane corresponding to 30× coverage) for each of the 4 clinical prostate cancer samples (see methods) and termed WGS ‘Gold Standard’ SNP data (WGS-GS). We found that ~ 95–96% of the SNPs detected in the spike-in WGS data (Additional file 1: Table 2; Additional file 2: Fig. 3) and ~ 55–57% of SNPs from WGBS data to be concordant with the WGS-GS data (Additional file 1: Table 3; Additional file 2: Fig. 3) indicating a higher degree of false positives called in the WGBS data, as previously reported [34]. However, we did find that there is a similar distribution of variant calls across all genomic features (Additional file 1: Table 4). We also found that WGBS detects a higher percentage of SNPs at regions with higher GC content (0.5–0.75), whereas WGS-GS detects a higher percentage of SNPs at regions with lower GC content (0.1–0.4) (Additional file 2: Fig. 4a). In addition, a higher percentage of SNPs were detected by WGS-GS near CpG sites which have high DNA methylation levels, (> 0.75) (Additional file 2: Fig. 4b), whereas WGBS detects a higher percentage of SNPs near CpG sites which are unmethylated or are lowly methylated, (< 0.5) (Additional file 2: Fig. 4b). Together our analyses indicate that integrated sequencing of WGBS and WGS libraries provides an efficient and cost-effective method to explore combinatorial analyses of genetic and epigenetic variations on one common technology platform.

### Correlation between library duplicate reads, spike-ins comparing HiSeq 2500 and HiSeq X Ten sequencing platforms

In general, we also observed that the duplicate read percentage for bisulphite libraries on the HiSeq X Ten were much higher for all than the duplicate reads observed for bisulphite libraries on the HiSeq 2500 platform (Tables 1, 4). To investigate this further, we compared the duplicate reads obtained from bisulphite libraries of cell line DNA samples sequenced on both the HiSeq 2500 and the HiSeq X Ten. Interestingly, for the same library preparation, we consistently obtained more duplicate reads on the HiSeq X Ten than the HiSeq 2500. For example, the duplicate read on the HiSeq 2500 for the bisulphite libraries (cell lines) was ~ 1.2–2.7 and ~ 15–18% on the HiSeq X Ten (Table 6); despite this, the coverage was consistently higher using the HiSeq X Ten.

**Table 6:** Comparison of duplicate reads obtained for the same libraries sequenced on both HiSeq 2500 and HiSeq X Ten

Library preparation method	Library preparation kit	Input amount (ng)	Sequencing platform	DNA samples	Raw reads	Duplicate reads (%)	Coverage	Ratio of CpG islands/shores
Post-BS	TruMethyl WG v1.9	200	HiSeq 2500 Rapid run^a^	1. Cell line (B80-T17-p12)	303298084	2.0	9.12×	1.0:1.1
				2. Cell line (B80-T17-p95)	412412960	2.7	12.40×	0.9:1.1
				3. Cell line (B80-T8-p8)	309366352	1.9	9.48×	1.0:1.1
				4. Cell line (B80-T8-p46)	337623632	2.2	10.20×	1.0:1.1
				5. Cell line (MCF7)	232184682	1.6	7.24×	1.0:1.1
				6. Cell line (TAMR)	28866392	1.2	6.66×	1.0:1.1
			HiSeq X Ten	1. Cell line (B80-T17-p12)	549,330,057	18	16.41×	1.0:1.1
				2. Cell line (B80-T17-p95)	623,032,192	18	18.83×	0.8:1.0
				3. Cell line (B80-T8-p8)	534,563,634	15	16.84×	1.0:1.1
				4. Cell line (B80-T8-p46)	551,950,690	16	17.24×	1.0:1.1
				5. Cell line (MCF7)	634,201,220	19	23.43×	0.9:1.0
				6. Cell line (TAMR)	571,714,843	15	20.48×	0.9:1.0

^a^One rapid run is two lanes

**Fig. 4:**
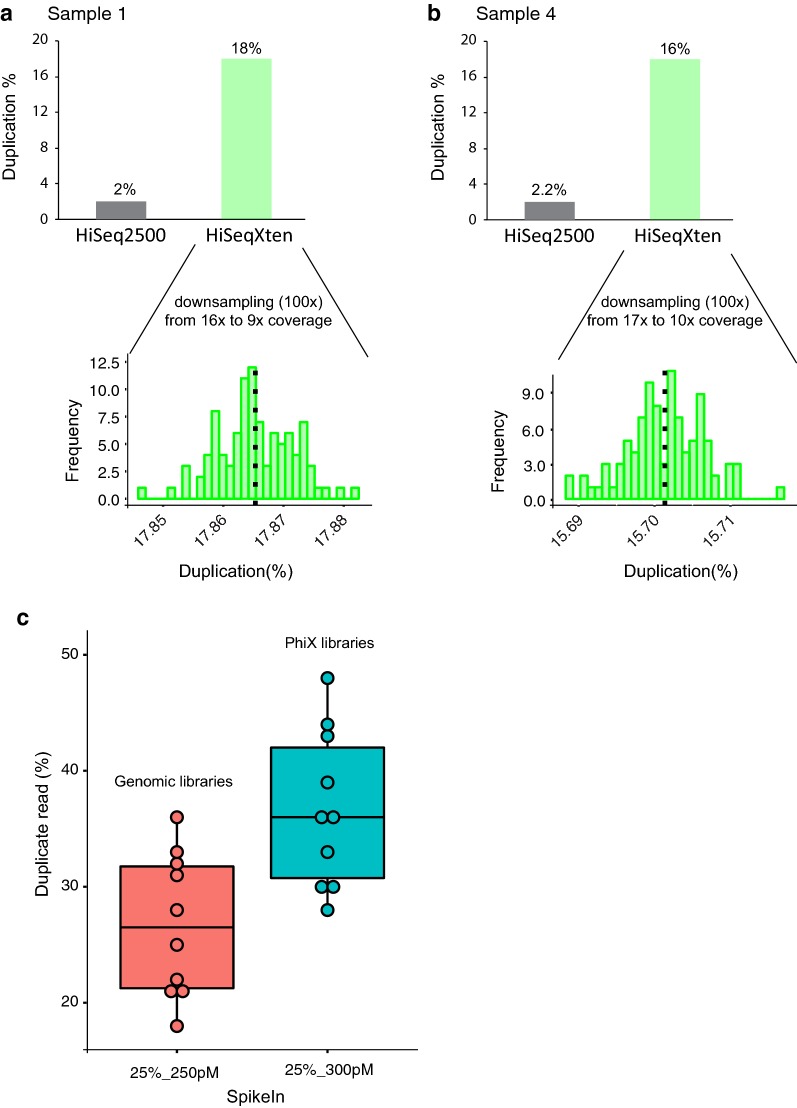
Correlation between duplicate reads, spike-ins and the sequencing platforms. **a**, **b** Plots showing the frequency of distribution of duplicate reads for two cell line DNA samples during down sampling of the raw reads from HiSeq X Ten to the number of raw reads obtained from HiSeq 2500. **c** Box plot showing the difference in duplicate percentage when the same set of ten bisulphite libraries were spiked with 25% of 250 pM genomic library and 25% of 300 pM PhiX library

To next determine if the higher coverage contributes towards the greater duplicate reads of the bisulphite libraries observed on the HiSeq X Ten, we measured the distribution of duplicate reads after randomly downsampling the number of raw reads obtained from the HiSeq X Ten platform to approximately the same number of raw reads obtained from the HiSeq 2500. Random downsampling was performed approximately 100 times, and the distribution of duplicate reads was estimated for two DNA samples (Fig. 4a, b). We observed that the distribution of duplicate reads for the bisulphite libraries on the HiSeq X Ten was similar across the hundred simulations (Fig. 4, b). For example, the distribution of percentage of duplicate reads in sample 1 ranged between 17.85 and 17.88% for different simulations and the frequency of such occurrence for each simulation is shown in the *y*-axis (Fig. 4a). These results support that the high duplicate reads observed on the HiSeq X Ten are a machine-generated artefact rather than an indication of library complexity.

We also observed that the rate of duplicate reads for the same bisulphite library was higher when the library was sequenced with the 300 pM PhiX spike compared to the 250 pM genomic spike (Fig. 4c; Table 4, for ten independent bisulphite libraries). Differences in duplicate read numbers for the same bisulphite library preparation with different spike-ins suggest that the duplicate reads are not due to PCR amplification bias inherent to the library, instead we surmise that they are due to the way the clusters are spatially distributed in the nanowells of the HiSeq X Ten’s patterned flow cells. This spatial distribution of the clusters could be dependent on the nature of spike-in DNA library loaded, its concentration and insert size.

### Comparison of overall coverage between HiSeq 2500 and HiSeq X Ten

**Fig. 5:**
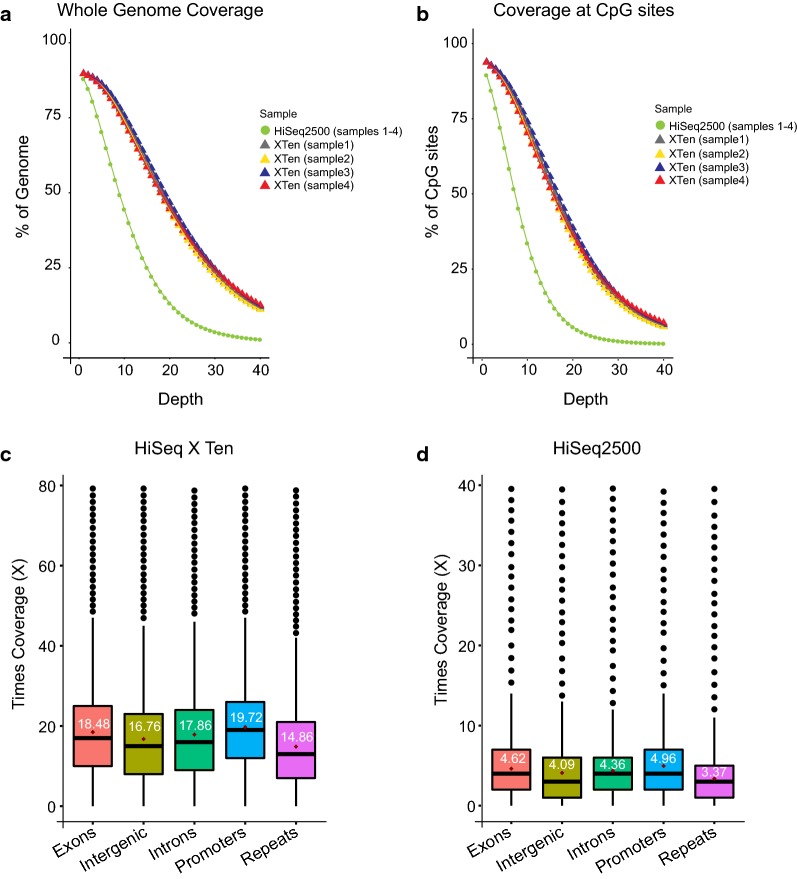
Coverage comparison between HiSeq 2500 and HiSeq X Ten. **a** Plot shows the fraction of genome covered at different depths for four samples sequenced together on one lane of the HiSeq 2500 versus when each of the samples is sequenced on a single lane of the HiSeq X Ten. The coverage plot for the HiSeq 2500 HO mode is the merged coverage obtained from multiplexing the four samples. **b** Plot shows the fraction of CpG sites covered at different depths when four clinical samples are sequenced together on one lane of the HiSeq 2500 versus when each of the samples is sequenced on a single lane of the HiSeq X Ten. **c**, **d** Box plot showing the coverage distribution across exons, intergenic regions, introns, promoter regions and repeat regions of the genome for a sample sequenced on one lane of HiSeq X Ten (**c**) and HiSeq 2500 (**d**)

Next, we compared the difference in overall genome coverage and individual CpG site coverage obtained from WGBS from four clinical samples prepared by the TruMethyl WG method, on the HiSeq 2500 High Output mode (HO) and on the HiSeq X Ten (Fig. 5a, b). The bisulphite libraries were all spiked with 25% of 300 pM PhiX for WGBS on the HiSeq X Ten and gave an overall coverage of ~ 16–20× per lane compared to ~ 8× coverage per lane on the HiSeq 2500. Figure 5a summarises the whole genome coverage plot for the four samples sequenced individually on single lanes of the HiSeq X Ten and merged coverage obtained from multiplexing the four samples on one lane of HiSeq 2500 high output mode. We found that almost 75% of the genome is covered at a depth of 10× from the HiSeq X Ten single lane sequencing, while only 40% of the genome is covered at a depth of ~ 10× from one lane of sequencing on the HiSeq 2500 (Fig. 5a). In addition, we assessed the coverage at individual CpG sites and found that only 30% of CpG sites were covered at a depth of ~ 10× from the HiSeq 2500 sequencing (Fig. 5b). However, ~ 70% of CpG sites were covered at a depth of ~ 10× from the HiSeq X Ten sequencing (Fig. 5b). We also compared the difference in coverage at specific genomic regions including, exons, intergenic regions, introns, promoters and repeat regions between the HiSeq 2500 platform and HiSeq X Ten platform for a clinical sample and a cell line (Fig. 5c, d, Additional file 2: Fig. 5a, b). We find that the HiSeq 2500 sequencing platform results in coverage ranging between 2.7× to 5.0× per lane across the different genomic regions, whereas the HiSeq X Ten platform results in coverage ranging between ~ 15× to 20× per lane across these same regions (Fig. 5c, d; Additional file 2: Fig. 5a, b).

### Comparison of library preparation methods on FFPET DNA on the HiSeq 2500 Platform

**Fig. 6:**
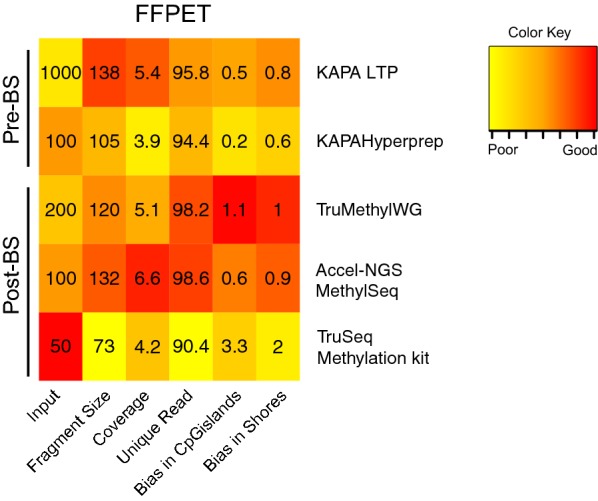
Comparing different library preparation methods using FFPET DNA. Cluster plot of sequencing output metrics obtained from different library preparation methods from FFPET DNA

Archival FFPET DNA is a valuable resource in cancer research to explore methylation alterations in cancer samples retrospectively; however, the DNA is generally degraded and genome-wide methylation analysis can be a challenge. In order to determine if FFPET DNA can be used for WGBS on the HiSeq X Ten platform, we first performed a comparison of two pre-BS and the three Post-BS methods on the HiSeq 2500 platform, using FFPET DNA, isolated from prostate cancer biopsies (Table 7). Since FFPET DNA is commonly degraded (< 300 bp), a comparison of the library preparation methods with varying size selection was not feasible. We found that for FFPET DNA the different library methods were fairly similar and gave lower coverage than for intact DNA, ranging from 3.9× (KAPA Hyperprep) to 6.6× coverage (Accel-NGS Methyl-Seq method) from two lanes (one rapid run on the HiSeq 2500) (Table 7; Fig. 6). Overall, with regard to input amount, fragment size and coverage, the Accel-NGS Methyl-Seq method performed the best using 100 ng of DNA and 132 bp fragment library size (Fig. 6). However, with regard to the bias ratio for representation of CpG islands and CpG shores, we found that the Accel-NGS Methyl-Seq method under represented CpG islands (ratio of 0.6:0.9), whereas the TruMethyl WG method showed good coverage across these CpG-rich features (ratio of 1.1:1) (Table 7). We therefore decided to test both the TruMethyl WG and Accel-NGS Methyl-Seq method for WGBS performance on the HiSeq X Ten.

**Table 7:** Comparison of different library preparation kits on FFPET using HiSeq 2500 Platform

Library preparation method	Library preparation kit	Input amount (ng)	Duplicate read (%)	Fragment size (stdev)	Coverage^a^	Ratio of bias in CpG island/shores
Pre-BS	KAPA LTP (Kapa Biosystems)	1000	4.2	138 bp (41 bp)	5.4×	0.5:0.8
	KAPA Hyperprep (Kapa Biosystems)	100	5.6	105 bp (49 bp)	3.9×	0.2:0.6
Post-BS	TruSeq DNA methylation (Illumina)	50	9.6	73 bp (37 bp)	4.2×	3.3:2.0
	TruMethyl WG v1.9 (Cambridge Epigenetix)	200	1.8	120 bp (49 bp)	5.1×	1.1:1.0
	Accel-NGS methyl-seq (Swift Biosciences)	100	1.4	132 bp (50 bp)	6.6×	0.6:0.9

^a^Two lanes (HiSeq 2500 rapid run)

### WGBS of FFPET DNA on the HiSeq X Ten

**Fig. 7:**
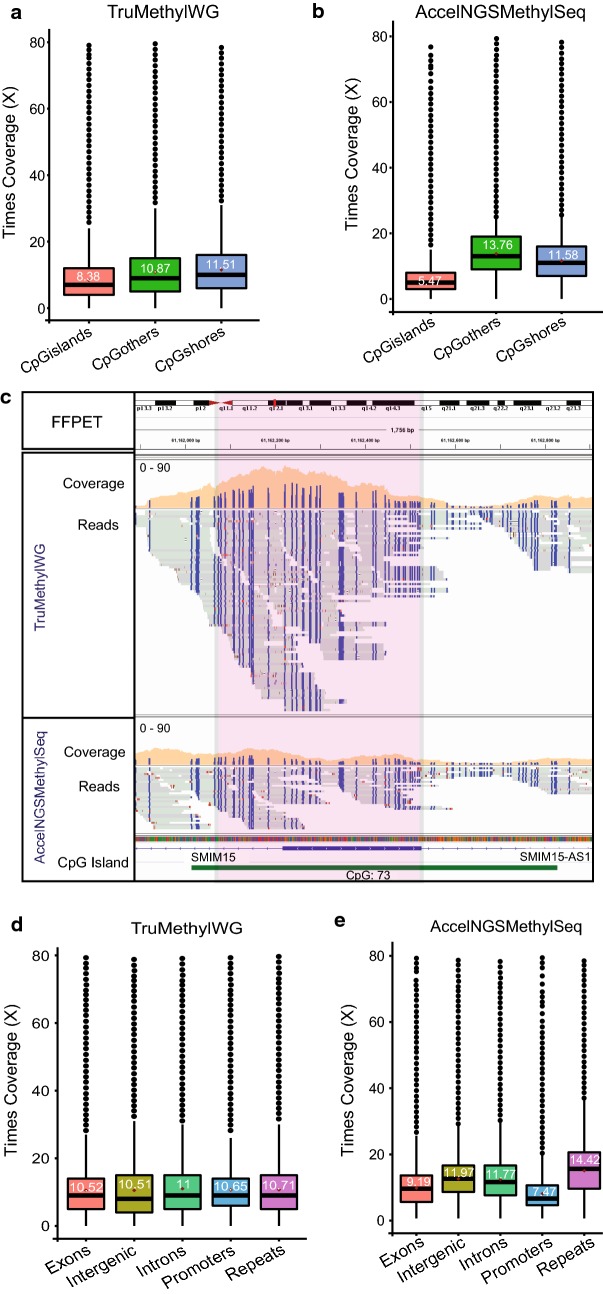
Difference in HiSeq X Ten coverage distribution for FFPET bisulphite library prepared from two methods. **a**, **b** Box plot showing the difference in coverage across CpG islands, CpG shores and other regions of the genome for TruMethyl WG (**a**) and Accel-NGS Methyl-Seq (**b**) methods, when sequenced on the HiSeq X Ten. **c** IGV plot showing the difference in distribution of reads for a FFPET library obtained from the TruMethyl WG method and Accel-NGS Methyl-Seq method across a CpG island. **d**, **e** Box plots showing the coverage distribution across exons, intergenic regions, introns, promoter regions and repeat regions of the genome for a FFPET library prepared by the TruMethyl WG (**d**) and Accel-NGS Methyl-Seq (**e**) methods and sequenced on one lane of HiSeq X Ten

We first compared WGBS coverage and bias outputs on the HiSeq X Ten from FFPET DNA using the TruMethyl WG and Accel-NGS Methyl-Seq method, with 25% of 300 pM PhiX as the spike-in concentration. We observed that the Accel-NGS Methyl-Seq method gave higher coverage (~ 13.05–13.97×) per lane and lower duplicate reads than the TruMethyl WG method (10.84–11.16×) for the same FFPET DNA (Table 8). However, as we found for intact DNA, the ratio of representation of CpG islands and CpG shores is less biased using the TruMethyl WG method, for example, 0.4:0.9 for Accel-NGS Methyl-Seq versus 0.8:1.1 for TruMethyl WG (Table 8). We further confirmed the apparent bias by determining the average coverage across CpG islands, CpG shores and other regions of the genome for WGBS data obtained from both the TruMethyl WG method and the Accel-NGS Methyl-Seq method (Fig. 7a, b). We then investigated candidate CpG islands and compared the reads spanning the CpG islands, between the TruMethyl WG method and the Accel-NGS Methyl-Seq method (Fig. 7c; Additional file 2: Fig. 6a, b) We also computed the CpG coverage distribution across exons, intergenic regions, introns, promoters and repeat regions of the genome (Fig. 7d, e). Our results confirmed that the representation of reads across specific genomic features is less biased in the TruMethyl WG method. However, the Accel-NGS Methyl-Seq method represents a higher fraction of repeat regions than the genomic features (Fig. 7d, e).

**Table 8:** Comparison of coverage output of FFPET on the HiSeq X Ten using two library preparation kits

Library preparation method	Library preparation kit name	Input amount (ng)	DNA samples FFPET	Sequencing platform	Fragment size (stdev)	Duplicate read (%)	Coverage/lane	CpG island/shore^a^
Post-BS	Accel-NGS Methyl-Seq	100	1. Prostate normal(1601)	HiSeq X Ten	158 bp (51 bp)	13	13.05×	0.6:1.0
			2. Prostate cancer (1601)		177 bp (53 bp)	18	13.97×	0.4:0.9
	TruMethyl WG v1.9	200	1. Prostate normal (1601)		161 bp (81 bp)	23	11.16×	1.3:1.3
			2. Prostate cancer (1601)		174 bp (91 bp)	27	10.84×	0.8:1.1

^a^Ratio of coverage represented in the CpG islands and shores of the genome

### Comparison of overall methylation correlation for intact genomic DNA and FFPET between HiSeq 2500 and HiSeq X Ten

**Fig. 8:**
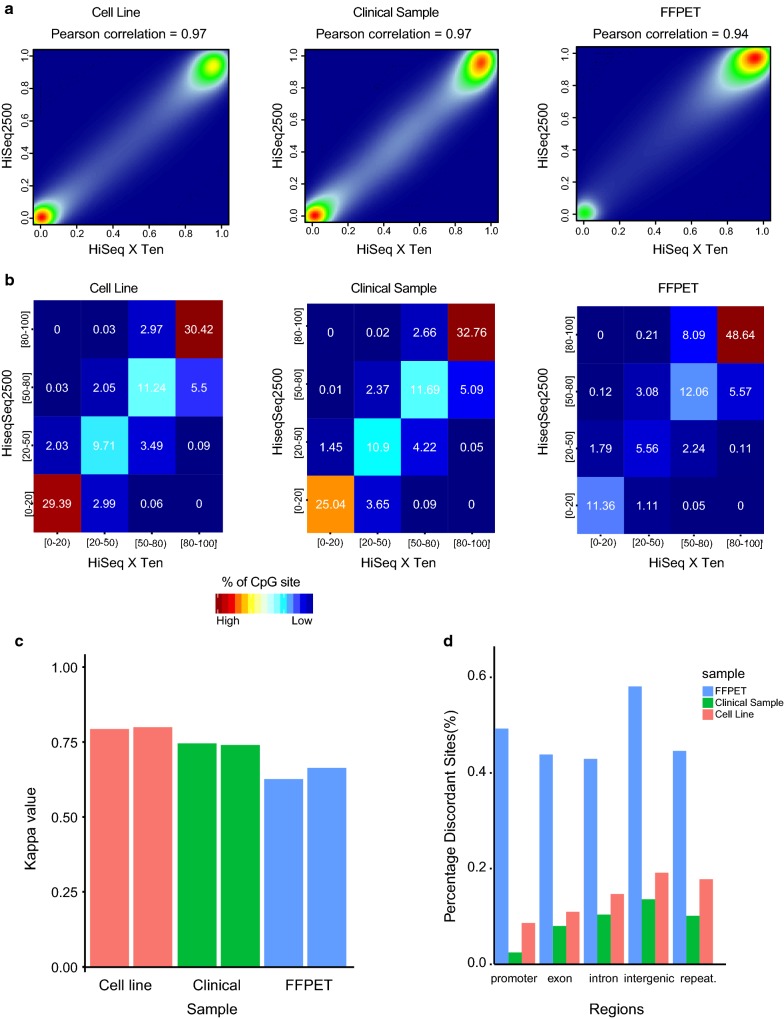
Comparison of methylation correlation between HiSeq 2500 and HiSeq X Ten. **a** Correlation plots of methylation levels obtained from a cell line, a clinical sample and a FFPET sample sequenced on the Hiseq 2500 versus HiSeq X Ten (Pearson *r* > 0.94). **b** Correlation of methylation values obtained from HiSeq 2500 and HiSeq X Ten for a cell line, clinical sample and a FFPET sample after grouping them into four bins of methylation percentages. **c** Average kappa values for six sample pairs, including two cell lines, two clinical samples and two FFPE samples compared between the HiSeq 2500 and HiSeq X Ten platform. **d** Bar plot showing the distribution of percentage of discordant sites across the genome for a cell line, clinical sample and a FFPET sample

We observed that despite differences in the chemistry between the two Illumina sequencing platforms and resulting genome-wide coverage per lane of sequencing, there is a remarkable correlation between the methylation calls obtained from the HiSeq 2500 and HiSeq X Ten, as demonstrated using DNA from a cell line, clinical sample and FFPET sample (Pearson *r *> 0.94) (Fig. 8a). We next classified CpG sites into four bins based on their methylation level percentage, namely under-methylated (0–20%), low methylation (20–50%), intermediate methylation (50–80%) and high methylation (80–100%), and determined the percentage of CpG sites, in the different methylation bins between the two platforms (Fig. 8b). Again we found high concordance in methylation levels between both the HiSeq 2500 and the HiSeq X Ten platform. To further test the agreement between the methylation data at different bins of CpG methylation percentage, we used the Kappa statistics [35]. The average kappa values for 6 sample pairs, (two cell lines, two clinical samples and two FFPET samples), compared between the two platforms is > 0.75 for the cell lines, ~ 0.75 for the clinical samples and 0.6 for FFPET (Fig. 8c). These values indicate that there is minimal difference in methylation calls obtained from both platforms; 0.21% CpG sites discordant in cell line DNA, 0.17% in the clinical samples and 0.49% in the FFPET sample. We found that these discordant sites were distributed across genomic features, including promoter, exon, intron, intergenic and repeat regions for all three-sample types (Fig. 8d). The higher rate of discordance identified for FFPET DNA could relate to the smaller fragment size relative to the intact DNA samples.

## Discussion

Over the past decade large-scale genome-wide methylation studies have been performed primarily with the Illumina 450 K methylation array platform, RRBS technology or targeted approaches [36–38]. Though these profiling methods are relatively cost-effective, they can only assess less than 5% of the CpG sites across the genome and primarily are biased to CpG islands [39]. Therefore, there is a strong need to develop an economical method to allow genome-wide methylation analysis. Currently, WGBS provides the gold standard for methylome analysis at single base resolution. However, the high sequencing cost, considerable technical expertise required and associated bioinformatic challenges to process the data have limited the widespread application of WGBS. The advent of HiSeq X Ten has opened up possibilities of performing WGBS with greater coverage per lane at a relatively lower cost.

Here we compare the most common available library preparation methods and identify the most efficient method to prepare WGBS libraries to achieve the greatest sequencing coverage on the HiSeq X Ten (HCS 3.3.76). We provide strategic guidelines that routinely produce ~ 16–20× coverage per lane of sequencing for intact human DNA and ~ 10–13× coverage per lane from FFPET samples. We find that coverage is primarily influenced by library fragment size and the nature and amount of the spike-in DNA. First, we show that the library choice can influence coverage and this is due to the resulting library fragment size that is obtained, as increasing fragment size results in higher sequencing coverage. Even greater coverage is expected if 100 bp PE reads could be supported on the HiSeq X Ten, since the current requirement to use 150 bp PE reads means there is still considerable read wastage due to the smaller fragments generated after bisulphite treatment.

Second, we find that adding insufficient amounts of the spike-in library can lead to poor cluster passing filter of the bisulphite library leading to lower coverage. We identify an optimum amount of spike-in libraries both for PhiX and genomic DNA to achieve the best coverage for human bisulphite libraries. Even though PhiX resulted in more WGBS coverage per lane of sequencing, the advantage of spiking genomic DNA from the same sample into the sequencing run allowed for the potential to identify SNP variants along with DNA methylation calling and therefore reduces read wastage.

Finally, our study compares the coverage obtained for a human bisulphite library from one lane of HiSeq 2500 sequencing and one lane of HiSeq X Ten sequencing. We show there is a high concordance in methylation levels obtained from both the platforms. However, only 30% of the CpG sites are covered at a depth of 10× in sequencing from the HiSeq 2500 platform, and almost 70% of the CpG sites are covered at a depth of 10× in the sequencing from the HiSeq X Ten platform.

A summary of our workflow for achieving optimal coverage on the HiSeq X Ten for intact genomic DNA and FFPET DNA is shown in Additional file 2: Fig. 7a, b. This workflow can also be potentially transferred to the newer software version HCS 3.4.0.38 and the newer Illumina sequencing platform, NovaSeq, which uses patterned flow cell similar to the HiSeq X Ten. The applicability of WGBS on large-scale epigenome-wide mapping studies is on the rise, and different technologies will appear. In fact a study recently published in BioRxiv [40] has devised a completely new library preparation strategy involving tagmentation and bisulphite tagging and a sequencing approach using custom sequencing oligos to achieve high-coverage WGBS on the HiSeq X Ten. However, our results and observations provide an established protocol for generating good quality WGBS data of high coverage at a reasonable cost and in combination with WGS herald a new era for integrated genomic and methylation sequencing studies.

## Conclusions

In this study, we provide a systematic, efficient and complete approach to perform and analyse WGBS on the HiSeq X Ten. Our protocol allows for large-scale WGBS studies at reasonable processing time and cost on the HiSeq X Ten platform.

## Methods

### Cell lines

Prostate cancer cell line, LNCaP, and breast cancer cell line, MCF7, were obtained from the American Type Culture Collection (ATCC). The endocrine-resistant MCF7-derived cell line and tamoxifen-resistant (TAMR) were generated by the long-term culture of MCF7 cells in phenol red-free RPMI medium with 5% charcoal stripped FCS and 4-OH-tamoxifen [41]. The B80 cell lines used in Tables 3 and 6 are human mammary epithelial cells immortalised by simian virus 40 T-antigen [42]. All cell lines were cultured under recommended conditions at 37 °C and 5% CO_2_. Sample 27, from Table 4, is THP-1 cell line, and sample 28 is THP-1 treated with PMA. Human THP-1 cells were maintained in RPMI media supplemented with 10% (v/v) FCS, 0.05 mM 2-mercaptoethanol, 0.1 mg/ml penicillin/streptomycin and 2 mM l-glutamine. Differentiation of THP-1 into macrophages was performed by culturing the cells with 100 ng/ml phorbol-12-myristate 13-acetate (PMA) and 50 μM 2-mercaptoethanol for 48 h.

### DNA samples

Genomic DNA from cell lines was extracted using QIAmp DNA Mini kit (Qiagen, USA). DNA from blood samples and clinical prostate cancer samples were also extracted using the QIAmp DNA Mini kit (Qiagen, USA).

### Library preparation methods

#### Pre-BS library preparation method

The two pre-BS library preparation methods were the KAPA LTP library preparation method and the KAPA Hyper prep library preparation method, which was performed following the manufacturer’s protocol. For the size selection steps, with the KAPA LTP method, a dual size selection ratio of 0.5:1.0 followed by 0.7:1.0 was performed to get library size of approximately 400 bp. For a library size of 300 bp, we followed the manufacturer’s protocol where the size selection ratio recommended was 0.6:1 followed by 0.8:1. To achieve library fragment size bigger than 300 and 400 bp, fragments that were not bound to the beads were eluted out. With the KAPA Hyperprep method, the protocol by the manufacturer was followed except at the post-PCR clean-up size selection step, and two different AMPure XP bead ratios were used 0.75:1 and 0.85:1 to achieve library sizes of above 300 bp and above 200 bp, respectively. In both methods, the bisulphite conversion was performed using the EZ DNA Methylation Gold kit from Zymo Research.

#### Post-BS library preparation methods

The three post-BS library preparation methods used are the TruSeq DNA methylation kit from Illumina, the Accel-NGS Methyl-seq DNA library preparation from Swift Biosciences and the TruMethyl WG method from Cambridge Epigenetix (CEGX). The library preparation using the TruSeq DNA methylation kit was performed exactly as the manufacturer’s protocol. With the Accel-NGS Methyl-seq DNA library prep method, besides following the original protocol, a size selection of 0.85:1 was performed during the post-PCR SPRI clean-up step to achieve library size greater than 300 bp. For the TruMethyl WG method, library preparation and indexing were carried out as described in the CEGX TruMethyl WG user guide v2. Since the presence of even 5% of adaptor dimers in the library leads to a 60% contamination of adaptor dimer reads on the HiSeq X Ten, we further improved the protocol by adding an additional clean-up step in the end using AmPure Xp beads at a ratio of 50:50. For both TruSeq DNA methylation kit and the Accel-NGS Methyl-Seq kit, bisulphite conversion was performed using the EZ DNA Methylation Gold kit from Zymo Research. The TruMethyl WG method has its own bisulphite conversion process incorporated in the protocol.

### Library QC and quantification

All libraries were quantified using the Qubit and KAPA library quantification kit (KAPA Biosystems), and the library quality was assessed using the High-sensitivity DNA kit on the Agilent 2100 Bioanalyzer (Agilent, CA, USA). Paired-end 150 bp sequencing was performed for each library on the Illumina HiSeq 2500 and the HiSeq X Ten platform.

### CpG islands and CpG shores bias analysis

The genomic coordinates of CpG islands were obtained from http://hgdownload.cse.ucsc.edu/goldenpath/hg19/database/cpgIslandExt.txt.gz, and CpG shores are defined as the regions immediately flanking CpG islands up to 2kbp away from both sides of the islands. CpG others are all the hg19 human regions, which are not either in CpG islands or in CpG shores. To compute CpG coverage distribution of CpG islands, bedtools [44] was used to intersect genomic coordinates of CpG islands with coverage data of all hg19 CpG sites (~ 28 million CpGs). The same procedure was applied for both CpG shores and others. Finally, the coverage for the three classes was plotted as box plot using ggplot2 in R. With regard to the bias, a value < 0.75 indicates under-representation and > 1.2 indicates over-representation of reads across CpG islands and CpG shores.

### Exons, intron, promoter, repeats and intergenic coverage analysis

Annotation of known gene transcripts and repeat elements was obtained from UCSC (http://hgdownload.cse.ucsc.edu/goldenpath/hg19/database/knownGene.txt.gz, http://hgdownload.cse.ucsc.edu/goldenpath/hg19/database/rmsk.txt.gz). Genomic coordinates of repeat elements were obtained from rmsk.txt.gz file. Promoters are regions of non-repeat bases containing all bases ranging from upstream 1500 and downstream 500 base pairs of a known transcription start site (TSS) in knownGene.txt.gz and not overlapping with itself. Exons were non-repeat bases and obtained from knownGene.txt.gz and not overlapping with itself. Introns were non-repeat bases and bases that are flanked by two exons of a single transcript and no overlapping with itself. Finally, intergenic regions were identified as the remaining bases in the reference genome. For computing the CpG coverage distribution of exons, introns, promoter regions, repeats and intergenic regions, bedtools was used to intersect the relevant genomic coordinates with coverage data of all hg19 CpG sites. Finally, the coverage for all the five classes was plotted as box plots using ggplot2 in R.

### Estimating read duplication rate for WGBS data of lower coverage

Nearly 50% of reads were randomly withdrawn from bam files of HiSeq X Ten’s WGBS data of approximately 20× coverage by using samtools [43], and Picard tool 2.3.0 (http://broadinstitute.github.io/picard) was used to measure the read duplication rate of the down sampled bam file. This procedure was repeated 100 times to get the estimation of read duplication rate for the low-coverage WGBS data (5–10X).

### Evaluation of DNA methylation agreement between different platforms

Pairs of WGBS data in HiSeq 2500 and HiSeq X Ten with coverage of at least 15× for every CpG site were used to compute the Pearson correlation. These CpG pair sites were then plotted with smoothScatter in R. To further evaluate the DNA methylation agreement between the platforms, we binned these DNA methylation values into 4 bins of (0–20%), (20–50%), (50–80%) and (80–100%) as no methylation, low, intermediate and high DNA methylation, respectively. Then, these bins were plotted as a heatmap using gplots [44]. Finally, Kappa statistics was used as a measure of agreement between two different platforms [35] with function kappa2 (without weighted) in IRR package [47]. Kappa values from − 1 to 1 were assigned to the samples with values from + 0.0 to 0.2 indicating slight agreement, + 0.21–0.40 indicating fair agreement, + 0.41–0.60 indicating moderate agreement, + 0.61–0.80 indicating substantial agreement, + and 0.81 to 1.0 indicating perfect agreement.

### HiSeq X Ten WGBS processing pipeline

The increased coverage achieved on the HiSeq X Ten generates > 1 Tb of data per run. To process the data, we developed a new bioinformatics pipeline package, Meth10X, based on previously published P3BSseq package [45], to support the increased number of bisulphite reads and reduce the processing time significantly. The Meth10X pipeline takes raw bisulphite reads in fastq files and trims the adapters following the guide of pre kit. The trimmed fastq files are aligned with bwa-meth [46] to the reference genome. The generated bam files are marked with duplication and merged if necessary. Estimation of the duplication rate, coverage bias (genomic features) and methylation bias in reads is carried out to provide quality control. We also use Qualimap 2 [47] for further evaluation of the alignment, such as percentage of unmapped/mapped read metrics, mapping quality distribution, GC content distribution, insert size distribution and coverage distribution. The pipeline, Meth10X, can be accessed from github, https://github.com/luuloi/Meth10X. For DNA methylation calling, MethylDackel (https://github.com/dpryan79/MethylDackel) is used with three different cytosine context patterns (CG, CHH, CHG) and strand specificity. MethylDackel also gives more options to remove methylation bias by trimming bias before calling methylation levels.

### Estimating concordant SNPs from WGBS and WGS

For calling SNPs from WGBS, we incorporated Biscuit (https://github.com/zwdzwd/biscuit) within the Meth10X pipeline. For calling SNPs from spike-in WGS 50:50 mix data and WGS-GS, we used GenomeAnalysisTK [48] following GATK best practices for variant calling. All the SNP sets obtained were then filtered with at least 5× coverage and QUAL of SNP with at least 200.0 for spike-in WGS data and WGS-GS and 20.0 for WGBS data. The SNP concordance and discordance between pairs (spike-in WGS, WGS-GS and WGBS) were evaluated on the filtered vcf files by using hap.py package, a Haplotype VCF comparison tool (https://github.com/Illumina/hap.py). For assessing the GC content across the genome for both WGBS and WGS-GS data, we used bedtools and human genome hg19 to create GC content with 100 bp sliding window, called GC content track. We then overlapped the vcf files of SNP from WGBS and WGS-GS with the GC content track to get the GC content score for every SNP. Followed by this, the density distribution of GC content score of WGS-GS and WGBS was plotted by ggplot2 in R. To get the methylated and unmethylated ratio of CpG sites near to a SNP across the genome for both WGBS and WGS-GS data, we used bedtools to get the CpG site nearest to a SNP within 50 bp. Further, the CpG nearest track for both WGBS and WGS-GS was overlapped with the methylomes of the same sample to get the methylation ratio. The density distribution of methylation ratio for WGBS and WGS-GS was plotted by ggplot2 in R.

## Additional files

**Additional file 1: Table 1.** Comparison of number of SNPs called in both WGBS and spike-in WGS data at 13x and 30x coverage. **Table 2.** Comparison of number of SNPs concordant in spike-in WGS data with WGS-Gold Standard at 30x coverage. **Table 3.** Comparison of number of SNPs concordant in WGBS data with WGS-Gold Standard at 30x coverage. **Table 4.** Percentage of SNPs observed across different genomic contexts for WGS-GS and WGBS.

**Additional file 2.** Figure 1: Box plot showing the difference in coverage across CpG islands, CpG shores and other regions of the genome for each of the five library preparation methods compared. Figure 2 **a, b** Two representative examples of regions showing SNP in both the WGS and WGBS data of the same clinical sample. Figure 3 **a** Bar plot showing the percentage of SNPs from WGBS concordant in WGS-GS at ~ 26× coverage and the percentage of SNPs from spike-in WGS concordant in WGS-GS at 30× coverage. **b** A representative Venn diagram for one prostate cancer sample, 2ab showing the number of SNPs concordant at 26× coverage for WGBS and 30× coverage for spike-in WGS when compared with WGS-GS data. Figure 4 **a**, Plot showing the distribution of normalised frequency of number of SNPs called across regions of the genome with varying levels of GC content for WGBS and WGS-GS. **b** Plot showing the distribution of normalised frequency of number of SNPs called across regions of the genome with varying levels of methylation ratio of CpG sites nearest to a SNP within 50 bp. Figure 5 **a, b** Box plot showing the coverage distribution across exons, intergenic regions, introns, promoter regions and repeat regions of the genome for a cell line sequenced on one lane of HiSeq X Ten (a) and HiSeq 2500 (b). Figure 6 **a, b** Two examples showing the difference in distribution of reads for a FFPET library obtained from the TruMethyl WG method and Accel-NGS Methyl-Seq method across a CpG island. Figure 7 **a, b** Summary of workflow for achieving optimal coverage on the HiSeq X Ten for intact genomic DNA and FFPET DNA.

## Abbreviations

WGBS: whole genome bisulphite sequencing
WGS: whole genome sequencing
SNP: single nucleotide polymorphism

## References

[1] Meissner A (2008). Genome-scale DNA methylation maps of pluripotent and differentiated cells. Nature.

[2] Jones PA (2012). Functions of DNA methylation: islands, start sites, gene bodies and beyond. Nat Rev Genet.

[3] Feinberg AP (2007). Phenotypic plasticity and the epigenetics of human disease. Nature.

[4] Hovestadt V (2014). Decoding the regulatory landscape of medulloblastoma using DNA methylation sequencing. Nature.

[5] Kretzmer H (2015). DNA methylome analysis in Burkitt and follicular lymphomas identifies differentially methylated regions linked to somatic mutation and transcriptional control. Nat Genet.

[6] Gardiner-Garden M, Frommer M (1987). CpG islands in vertebrate genomes. J Mol Biol.

[7] Zhu J (2008). On the nature of human housekeeping genes. Trends Genet.

[8] Deaton AM, Bird A (2011). CpG islands and the regulation of transcription. Genes Dev.

[9] Feinberg AP, Vogelstein B (1983). Hypomethylation distinguishes genes of some human cancers from their normal counterparts. Nature.

[10] Cheah MS, Wallace CD, Hoffman RM (1984). Hypomethylation of DNA in human cancer cells: a site-specific change in the c-myc oncogene. J Natl Cancer Inst.

[11] Brunner AL (2009). Distinct DNA methylation patterns characterize differentiated human embryonic stem cells and developing human fetal liver. Genome Res.

[12] Brinkman AB (2010). Whole-genome DNA methylation profiling using MethylCap-seq. Methods.

[13] Gu H (2010). Genome-scale DNA methylation mapping of clinical samples at single-nucleotide resolution. Nat Methods.

[14] Serre D, Lee BH, Ting AH (2010). MBD-isolated genome sequencing provides a high-throughput and comprehensive survey of DNA methylation in the human genome. Nucleic Acids Res.

[15] Lister R (2009). Human DNA methylomes at base resolution show widespread epigenomic differences. Nature.

[16] Bibikova M (2009). Genome-wide DNA methylation profiling using Infinium (R) assay. Epigenomics.

[17] Bibikova M (2011). High density DNA methylation array with single CpG site resolution. Genomics.

[18] Pidsley R (2016). Critical evaluation of the illumina METHYLATIONEPIC BeadChip microarray for whole-genome DNA methylation profiling. Genome Biol.

[19] Zhou W, Laird PW, Shen H (2017). Comprehensive characterization, annotation and innovative use of Infinium DNA methylation BeadChip probes. Nucleic Acids Res.

[20] Suzuki MM, Bird A (2008). DNA methylation landscapes: provocative insights from epigenomics. Nat Rev Genet.

[21] Bibikova M, Fan JB (2010). Genome-wide DNA methylation profiling. Wiley Interdiscip Rev Syst Biol Med.

[22] Laird PW (2010). Principles and challenges of genomewide DNA methylation analysis. Nat Rev Genet.

[23] Beck S (2010). Taking the measure of the methylome. Nat Biotechnol.

[24] Bock C (2010). Quantitative comparison of genome-wide DNA methylation mapping technologies. Nat Biotechnol.

[25] Harris RA (2010). Comparison of sequencing-based methods to profile DNA methylation and identification of monoallelic epigenetic modifications. Nat Biotechnol.

[26] Schubeler D (2015). Function and information content of DNA methylation. Nature.

[27] Ziller MJ (2015). Coverage recommendations for methylation analysis by whole-genome bisulfite sequencing. Nat Methods.

[28] Jensen TJ (2015). Whole genome bisulfite sequencing of cell-free DNA and its cellular contributors uncovers placenta hypomethylated domains. Genome Biol.

[29] Jenkinson G (2017). Potential energy landscapes identify the information-theoretic nature of the epigenome. Nat Genet.

[30] Kulis M (2015). Whole-genome fingerprint of the DNA methylome during human B cell differentiation. Nat Genet.

[31] Durek P (2016). Epigenomic profiling of human CD4 + T cells supports a linear differentiation model and highlights molecular regulators of memory development. Immunity.

[32] Grunau C, Clark SJ, Rosenthal A (2001). Bisulfite genomic sequencing: systematic investigation of critical experimental parameters. Nucleic Acids Res.

[33] Miura F (2012). Amplification-free whole-genome bisulfite sequencing by post-bisulfite adaptor tagging. Nucleic Acids Res.

[34] Gao S (2015). BS-SNPer: SNP calling in bisulfite-seq data. Bioinformatics.

[35] McHugh ML (2012). Interrater reliability: the kappa statistic. Biochem Med (Zagreb).

[36] Pei L (2012). Genome-wide DNA methylation analysis reveals novel epigenetic changes in chronic lymphocytic leukemia. Epigenetics.

[37] Zouridis H (2012). Methylation subtypes and large-scale epigenetic alterations in gastric cancer. Sci Transl Med.

[38] El Hajj N (2017). DNA methylation signatures in cord blood of ICSI children. Hum Reprod.

[39] Stirzaker C (2014). Mining cancer methylomes: prospects and challenges. Trends Genet.

[40] Suzuki M, Liao W, Wos F, Johnston AD, DeGrazia J, Ishii J, Bloom T, Zody MC, Germer S, Greally JM. Whole genome bisulphite sequencing using the illumina Hiseq X system. BioRxiv preprint; 2017.

[41] Stone A (2015). DNA methylation of oestrogen-regulated enhancers defines endocrine sensitivity in breast cancer. Nat Commun.

[42] Toouli CD (2002). Comparison of human mammary epithelial cells immortalized by simian virus 40 T-antigen or by the telomerase catalytic subunit. Oncogene.

[43] Li H (2009). The sequence alignment/map format and SAMtools. Bioinformatics.

[44] Warnes GR, Bolker B. gplots: various R programming tools for plotting data. R package version 2.6.0, 2012.

[45] Luu PL (2017). P3BSseq: parallel processing pipeline software for automatic analysis of bisulfite sequencing data. Bioinformatics.

[46] Pedersen BS, Eyring K, De S, Yang IV, Schwartz DA. Fast and accurate alignment of long bisulfite-seq reads. ArXiv, 2014. 1401.1129v2.

[47] Okonechnikov K, Conesa A, García-Alcalde F (2015). Qualimap 2: advanced multi-sample quality control for high-throughput sequencing data. Bioinformatics.

[48] Van der Auwera GA (2013). From FastQ data to high confidence variant calls: the genome analysis toolkit best practices pipeline. Curr Protoc Bioinf.

