# Supplementary material for: Guidelines for whole genome bisulphite sequencing of intact and FFPET DNA on the Illumina HiSeq X Ten
Source: Epigenetics Chromatin. 2018 May 28;11:24. doi: 10.1186/s13072-018-0194-0 (PMC5971424; doi:10.1186/s13072-018-0194-0)
Supplement: Supplementary file 1 — Additional file 1: Table 1. Comparison of number of SNPs called in both WGBS and spike-in WGS data at 13x and 30x coverage. Table 2. Comparison of number of SNPs concordant in spike-in WGS data with WGS-Gold Standard at 30x coverage. Table 3. Comparison of number of SNPs concordant in WGBS data with WGS-Gold Standard at 30x coverage. Table 4. Percentage of SNPs observed across different genomic contexts for WGS-GS and WGBS. [file 13072_2018_194_MOESM1_ESM.pdf]

Supplementary Table 1: Comparison of number of SNPs called in both WGBS and spike-in WGS data at 13x and 30x coverage							
**Clinical Samples**	**Coverage**	**Total SNPs in WGS***	**Total SNPs in WGBS**	**Total number of Concordant SNPs**	**Total number of discordant SNPs in WGS***	**Total number of discordant SNPs in WGBS**	**Percentage of SNPs concordant in WGBS**
1a. Prostate cancer DNA (5287)	13x	2887873	2628374	1082941	1804932	1545433	41%
1b. Prostate cancer DNA (5287)		2931260	2602234	1091587	1839673	1510647	42%
2a. Prostate cancer DNA (5060)		2986340	2542079	1138285	1848055	1403794	45%
2b. Prostate cancer DNA (5060)		3009483	2511796	1133450	1876033	1378346	45%
3a. Prostate cancer DNA (13179)		2999151	2531518	1014533	1984618	1516985	40%
3b. Prostate cancer DNA (13179)		3028694	2442123	971701	2056993	1470422	40%
4a.Prostate cancer DNA (10738)		3092800	2583936	1130536	1962264	1453400	44%
4b.Prostate cancer DNA (10738)		3079779	2515847	1081888	1997891	1433959	43%
1ab. Prostate cancer DNA (5287)	30x	3416908	3152836	1639163	1777745	1513673	52%
2ab. Prostate cancer DNA (5060)		3455216	3190508	1685499	1769717	1505009	53%
3ab. Prostate cancer DNA (13179)		3468051	3085112	1573872	1894179	1511240	51%
4ab.Prostate cancer DNA (10738)		3518646	3143760	1671229	1847417	1472531	53%

* = spike-in WGS data

Supplementary Table 2: Comparison of number of SNPs concordant in spike-in WGS data with WGS-Gold Standard at 30x coverage							
**Clinical Samples**	**Coverage**	**Total SNPs in WGS-GS**	**Total SNPs in WGS***	**Total number of Concordant SNPs in WGS***	**Total number of discordant SNPs in WGS-GS**	**Total number of discordant SNPs in WGS***	**Percentage of SNPs concordant in WGS***
1ab. Prostate cancer DNA (5287)	30x	3662735	3416912	3246715	416020	170197	95%
2ab. Prostate cancer DNA (5060)		3654606	3455225	3291601	363005	163624	95%
3ab. Prostate cancer DNA (13179)		3659759	3468053	3302942	356817	165111	95%
4ab.Prostate cancer DNA (10738)		3692194	3518653	3367239	324955	151414	96%

* = spike-in WGS data

WGS-GS = WGS Gold standard

Supplementary Table 3: Comparison of number of SNPs concordant in WGBS data with WGS-Gold Standard at 30x coverage							
**Clinical Samples**	**Coverage**	**Total SNPs in WGS-GS**	**Total SNPs in WGBS**	**Total number of Concordant SNPs in WGBS**	**Total number of discordant SNPs in WGS-GS**	**Total number of discordant SNPs in WGBS**	**Percentage of SNPs concordant in WGBS**
1ab. Prostate cancer DNA (5287)	30x	3692194	3152836	1809494	1853241	1343342	57%
2ab. Prostate cancer DNA (5060)		3659759	3190508	1820002	1834604	1370506	57%
3ab. Prostate cancer DNA (13179)		3654606	3085112	1691494	1968265	1393618	55%
4ab.Prostate cancer DNA (10738)		3662735	3143760	1781298	1910896	1362462	57%

* = spike-in WGS data

WGS-GS = WGS Gold standard

Supplementary Table 4: Percentage of SNPs observed across different genomic contexts for WGS-GS and WGBS

Clinical Samples	Genomic Context	WGS-GS_Count	WGS-GS_Total	SNP Percentage (WGS-GS)	WGBS_Count	WGBS_Total	SNP Percentage (WGBS)
1a. Prostate cancer DNA (5287)	CpGs	578759	4875955	11.9	360010	3153015	11.4
2a. Prostate cancer DNA (5060)	CpGs	577629	4852201	11.9	367457	3190559	11.5
3a. Prostate cancer DNA (13179)	CpGs	579587	4863240	11.9	352144	3085162	11.4
4a.Prostate cancer DNA (10738)	CpGs	585312	4936223	11.9	360949	3143812	11.5
1a. Prostate cancer DNA (5287)	CpG Islands	39621	4875955	0.8	32121	3153015	1
2a. Prostate cancer DNA (5060)	CpG Islands	38039	4852201	0.8	30083	3190559	0.9
3a. Prostate cancer DNA (13179)	CpG Islands	38823	4863240	0.8	30044	3085162	1
4a.Prostate cancer DNA (10738)	CpG Islands	39562	4936223	0.8	31286	3143812	1
1a. Prostate cancer DNA (5287)	CpG Shores	166992	4875955	3.4	116880	3153015	3.7
2a. Prostate cancer DNA (5060)	CpG Shores	164639	4852201	3.4	116576	3190559	3.7
3a. Prostate cancer DNA (13179)	CpG Shores	167079	4863240	3.4	113890	3085162	3.7
4a.Prostate cancer DNA (10738)	CpG Shores	171328	4936223	3.5	116461	3143812	3.7
1a. Prostate cancer DNA (5287)	Intergenic	868471	4875955	17.8	586988	3153015	18.6
2a. Prostate cancer DNA (5060)	Intergenic	863861	4852201	17.8	593473	3190559	18.6
3a. Prostate cancer DNA (13179)	Intergenic	870706	4863240	17.9	579247	3085162	18.8
4a.Prostate cancer DNA (10738)	Intergenic	876643	4936223	17.8	585436	3143812	18.6
1a. Prostate cancer DNA (5287)	Repeat	2877330	4875955	59	1779417	3153015	56.4
2a. Prostate cancer DNA (5060)	Repeat	2854186	4852201	58.8	1799786	3190559	56.4
3a. Prostate cancer DNA (13179)	Repeat	2859690	4863240	58.8	1729885	3085162	56.1
4a.Prostate cancer DNA (10738)	Repeat	2909793	4936223	58.9	1770205	3143812	56.3
1a. Prostate cancer DNA (5287)	Exon	102115	4875955	2.1	77363	3153015	2.5
2a. Prostate cancer DNA (5060)	Exon	101990	4852201	2.1	76848	3190559	2.4
3a. Prostate cancer DNA (13179)	Exon	102834	4863240	2.1	75539	3085162	2.4
4a.Prostate cancer DNA (10738)	Exon	103905	4936223	2.1	76745	3143812	2.4
1a. Prostate cancer DNA (5287)	Intron	1059981	4875955	21.7	732206	3153015	23.2
2a. Prostate cancer DNA (5060)	Intron	1064810	4852201	21.9	743666	3190559	23.3
3a. Prostate cancer DNA (13179)	Intron	1062676	4863240	21.9	722819	3085162	23.4
4a.Prostate cancer DNA (10738)	Intron	1079369	4936223	21.9	734366	3143812	23.4
1a. Prostate cancer DNA (5287)	Promoter	43084	4875955	0.9	34476	3153015	1.1
2a. Prostate cancer DNA (5060)	Promoter	42529	4852201	0.9	34408	3190559	1.1
3a. Prostate cancer DNA (13179)	Promoter	43332	4863240	0.9	33624	3085162	1.1
4a.Prostate cancer DNA (10738)	Promoter	43226	4936223	0.9	34682	3143812	1.1
