# Supplementary material for: Guidelines for whole genome bisulphite sequencing of intact and FFPET DNA on the Illumina HiSeq X Ten
Source: Epigenetics Chromatin. 2018 May 28;11:24. doi: 10.1186/s13072-018-0194-0 (PMC5971424; doi:10.1186/s13072-018-0194-0)

Supplementary Figure 1

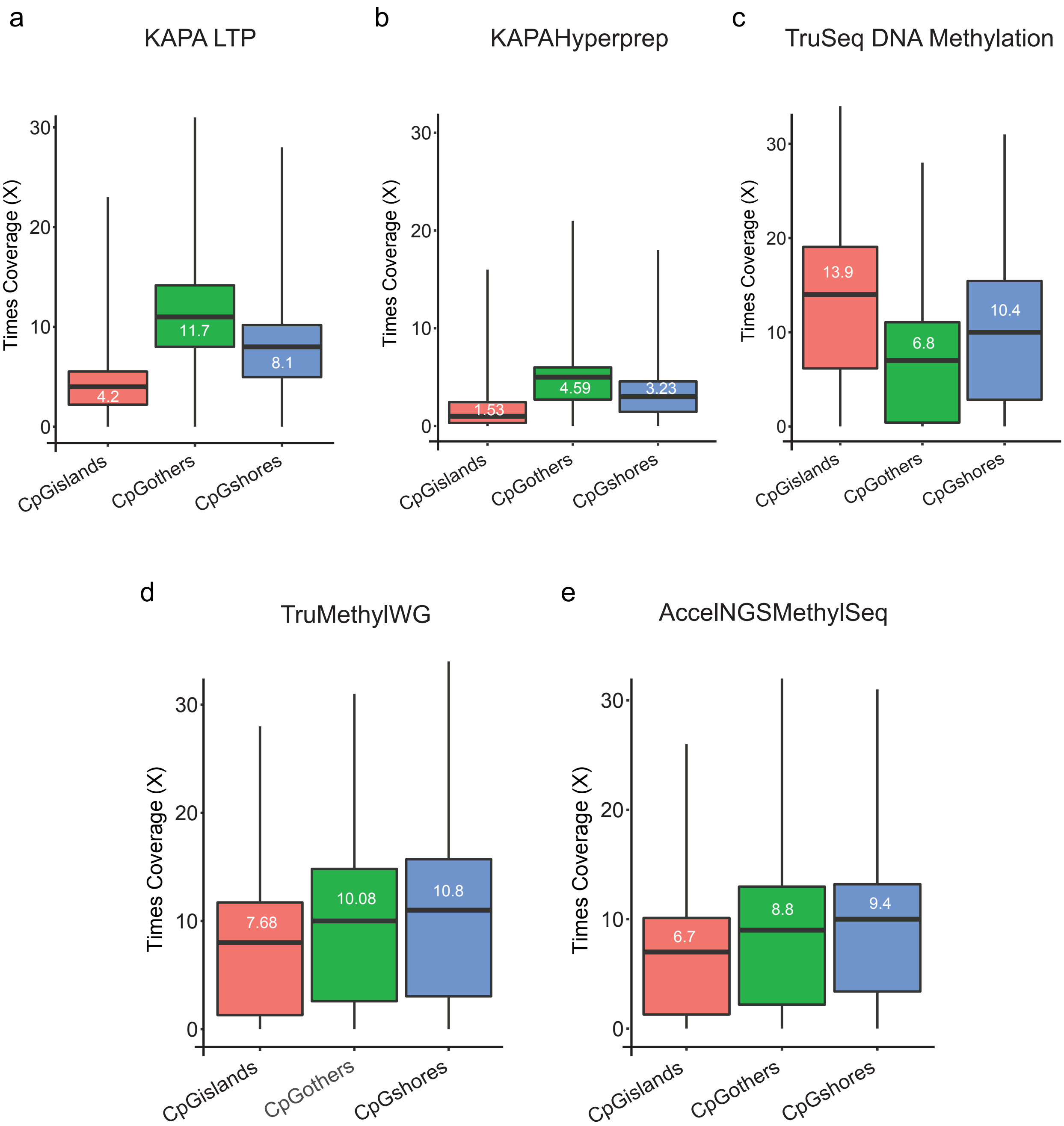

Supplementary Figure 2

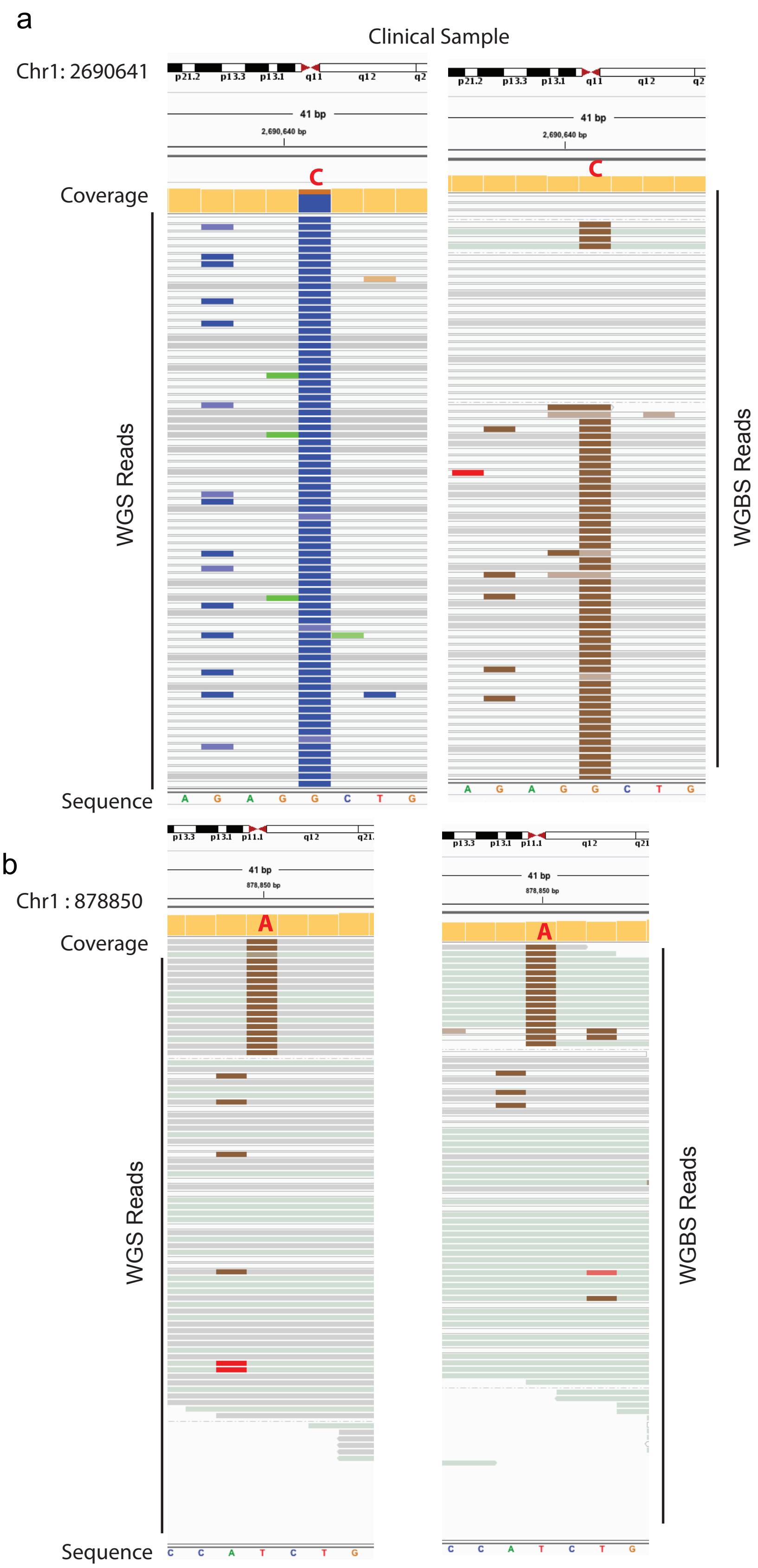

Supplementary Figure 3

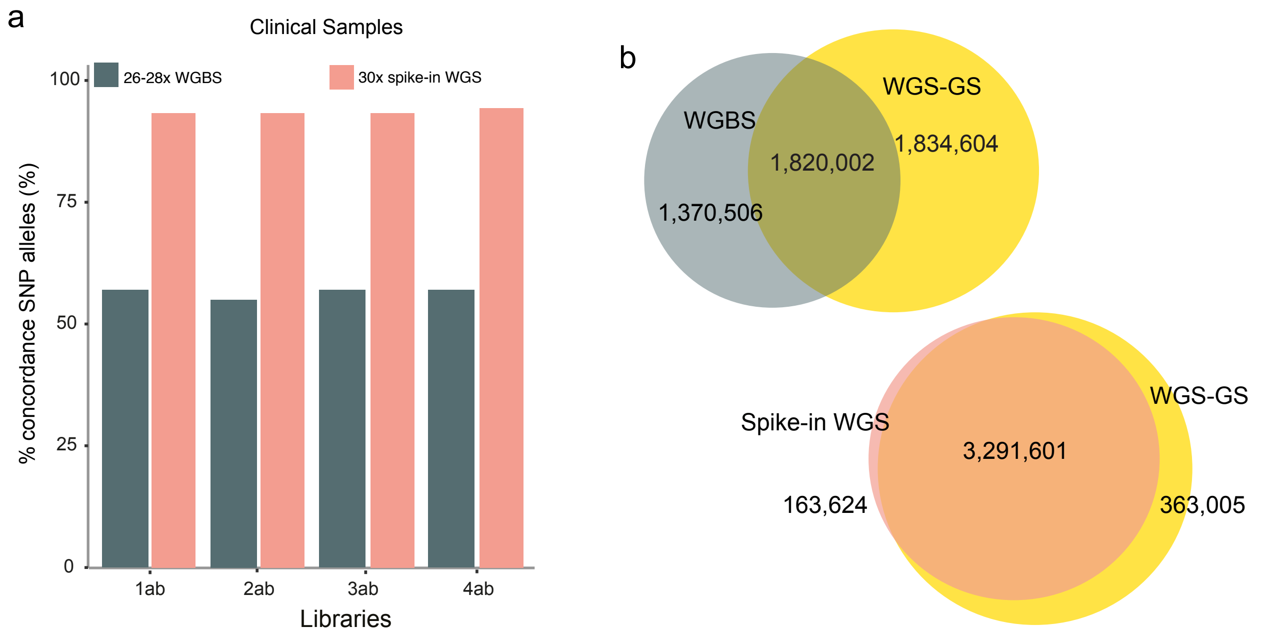

Supplementary Figure 4

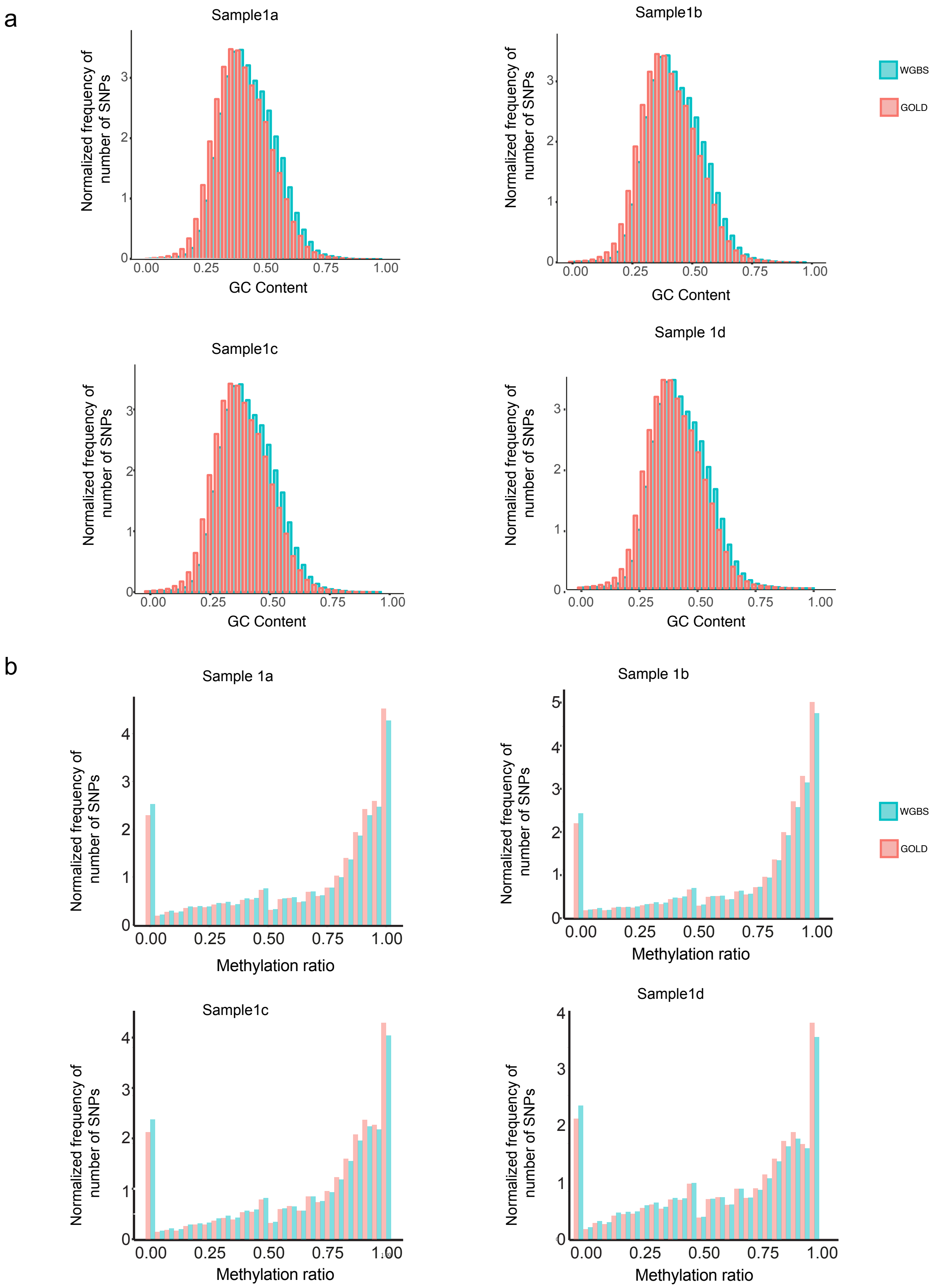

Supplementary Figure 5

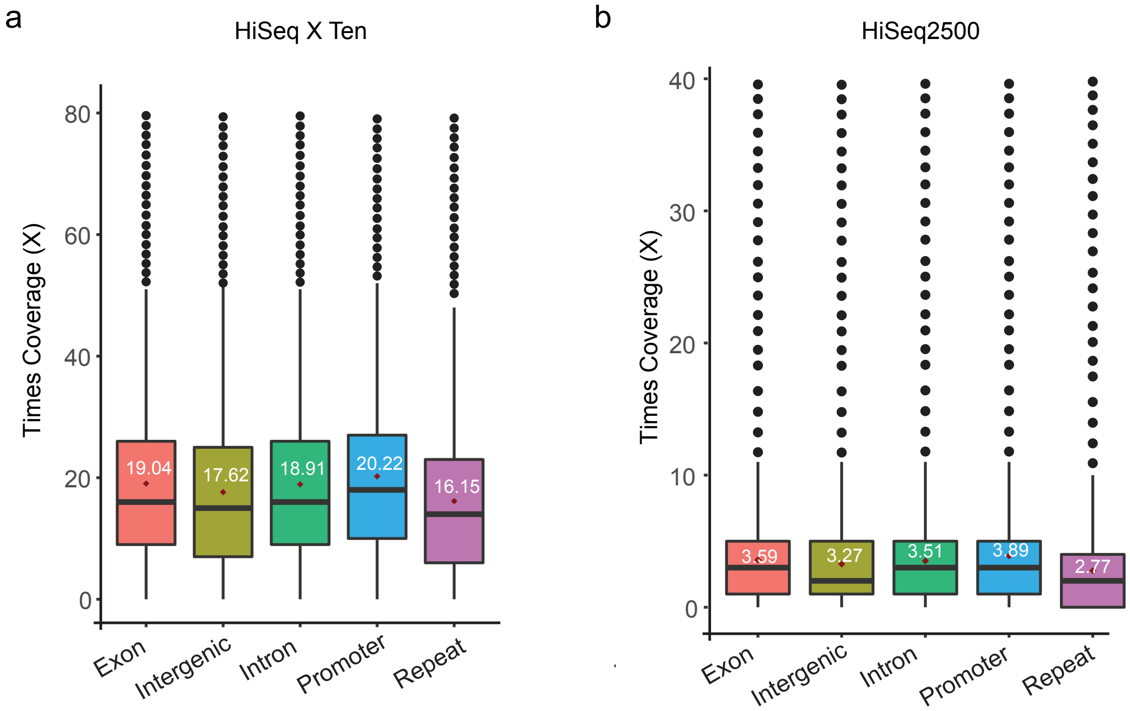

Supplementary Fig 6

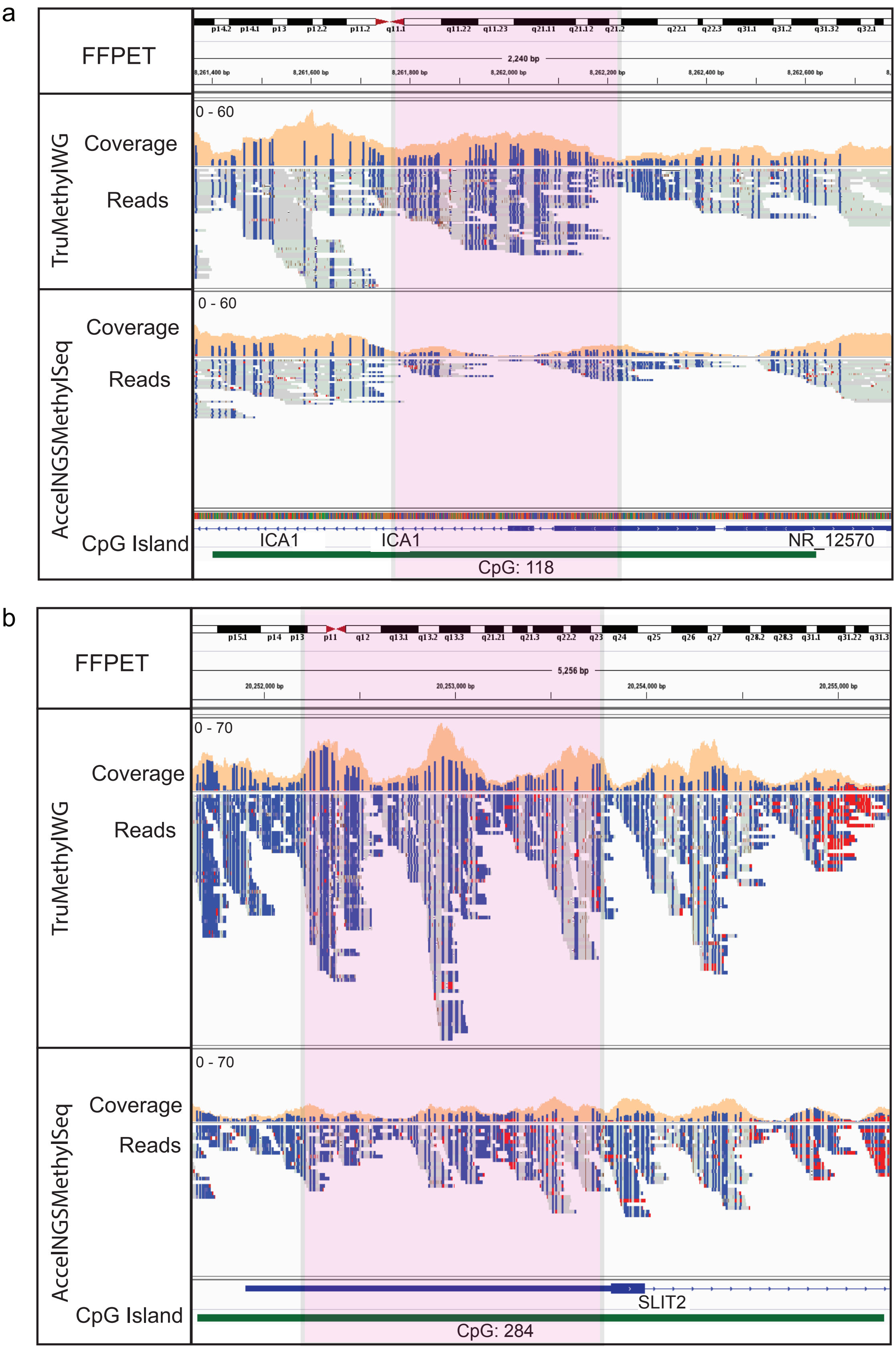

Supplementary Figure 7a

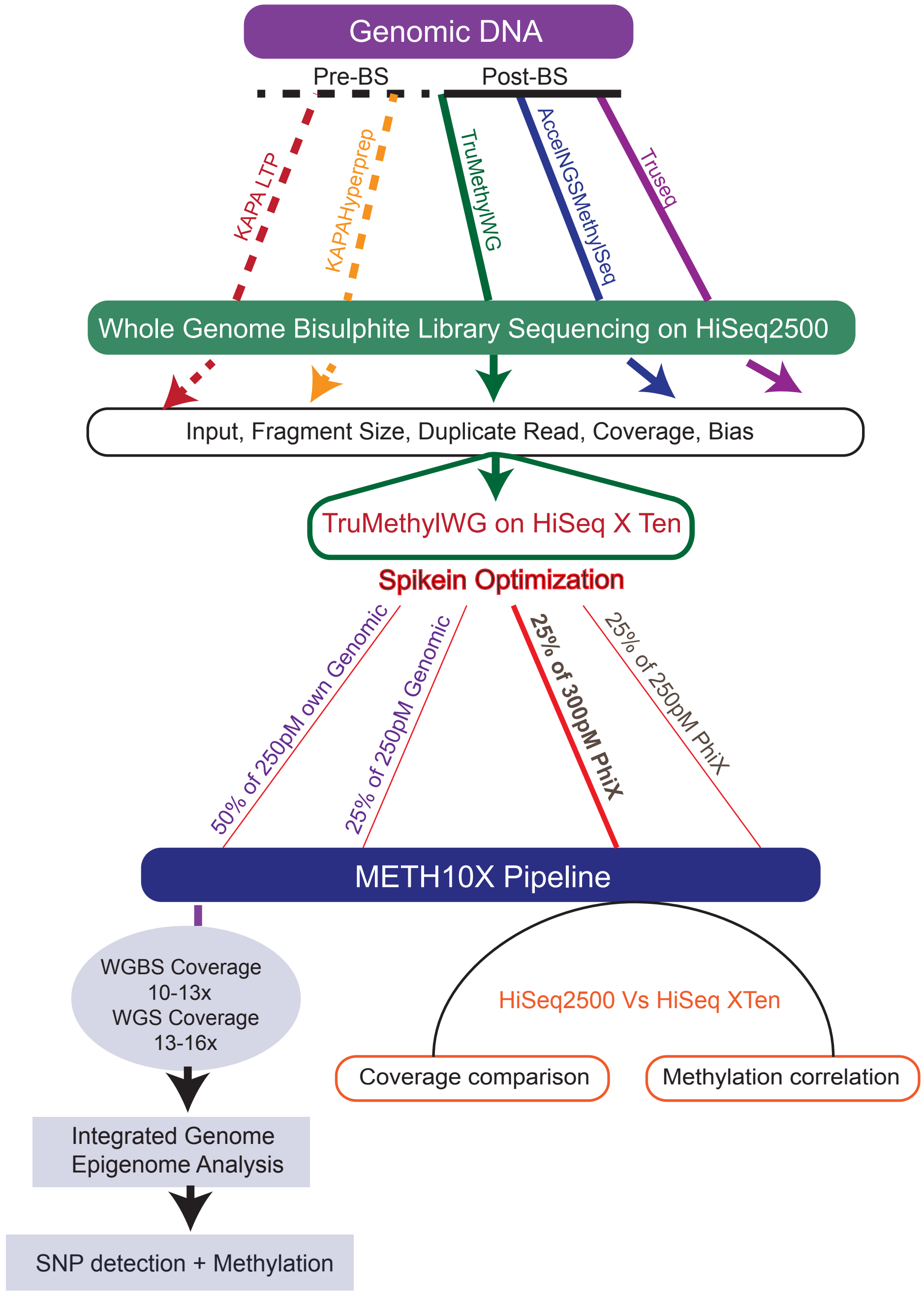

Supplementary Figure 7b

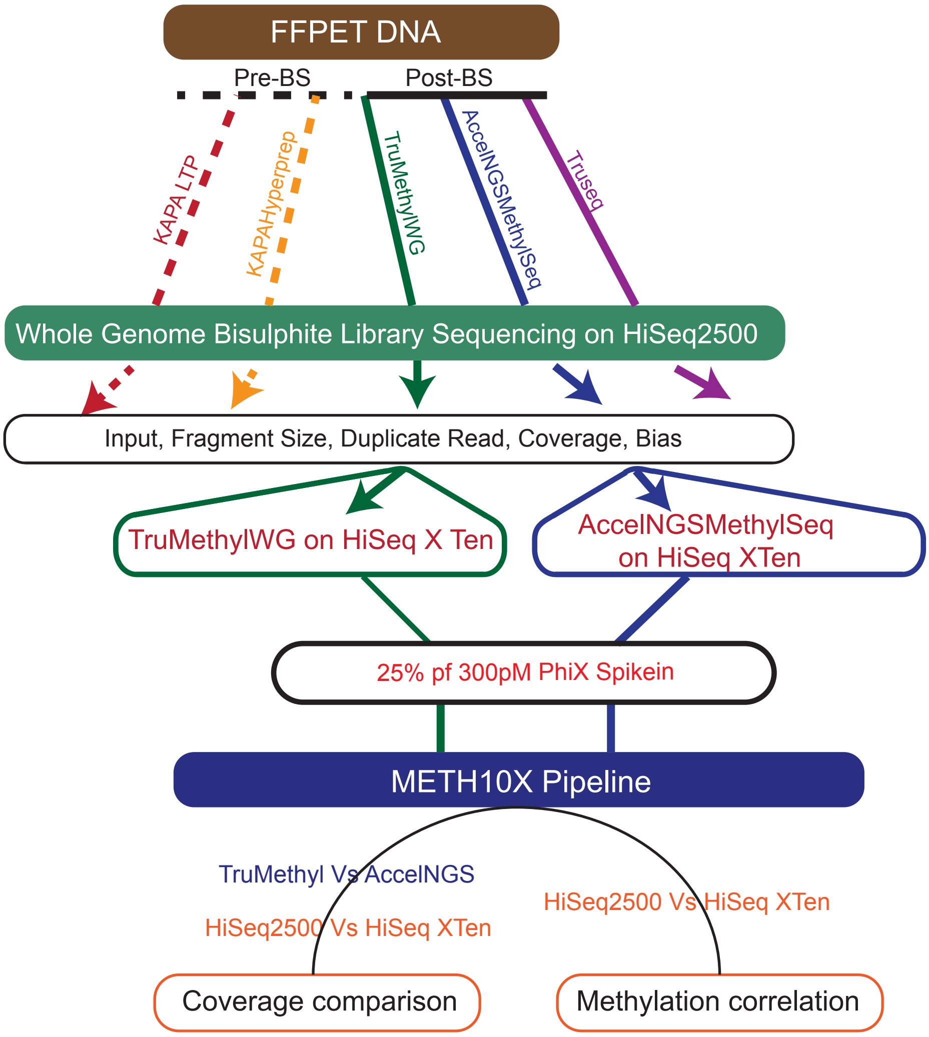

Supplement: Supplementary file 2 — Additional file 2. Figure 1: Box plot showing the difference in coverage across CpG islands, CpG shores and other regions of the genome for each of the five library preparation methods compared. Figure 2 a, b Two representative examples of regions showing SNP in both the WGS and WGBS data of the same clinical sample. Figure 3 a Bar plot showing the percentage of SNPs from WGBS concordant in WGS-GS at ~ 26× coverage and the percentage of SNPs from spike-in WGS concordant in WGS-GS at 30× coverage. b A representative Venn diagram for one prostate cancer sample, 2ab showing the number of SNPs concordant at 26× coverage for WGBS and 30× coverage for spike-in WGS when compared with WGS-GS data. Figure 4 a, Plot showing the distribution of normalised frequency of number of SNPs called across regions of the genome with varying levels of GC content for WGBS and WGS-GS. b Plot showing the distribution of normalised frequency of number of SNPs called across regions of the genome with varying levels of methylation ratio of CpG sites nearest to a SNP within 50 bp. Figure 5 a, b Box plot showing the coverage distribution across exons, intergenic regions, introns, promoter regions and repeat regions of the genome for a cell line sequenced on one lane of HiSeq X Ten (a) and HiSeq 2500 (b). Figure 6 a, b Two examples showing the difference in distribution of reads for a FFPET library obtained from the TruMethyl WG method and Accel-NGS Methyl-Seq method across a CpG island. Figure 7 a, b Summary of workflow for achieving optimal coverage on the HiSeq X Ten for intact genomic DNA and FFPET DNA. [file 13072_2018_194_MOESM2_ESM.pdf]
